# Composite Track-Etched Membranes: Synthesis and Multifaced Applications

**DOI:** 10.3390/polym16182616

**Published:** 2024-09-15

**Authors:** Anastassiya A. Mashentseva, Duygu S. Sutekin, Saniya R. Rakisheva, Murat Barsbay

**Affiliations:** 1 The Institute of Nuclear Physics of the Republic of Kazakhstan, Almaty 050032, Kazakhstan; 2 Department of Nuclear Physics, New Materials, and Technologies, L. N. Gumilyov Eurasian National University, Astana 010008, Kazakhstan; saniya.rakisheva58@gmail.com; 3 Department of Chemistry, Hacettepe University, Ankara 06800, Turkey; duygu@hacettepe.edu.tr

**Keywords:** composite track-etched membranes (CTeMs), track-etched membranes (TeMs), mmebrane technology, hybrid membranes, functional nanomaterials

## Abstract

Composite track-etched membranes (CTeMs) emerged as a versatile and high-performance class of materials, combining the precise pore structures of traditional track-etched membranes (TeMs) with the enhanced functionalities of integrated nanomaterials. This review provides a comprehensive overview of the synthesis, functionalization, and applications of CTeMs. By incorporating functional phases such as metal nanoparticles and conductive nanostructures, CTeMs exhibit improved performance in various domains. In environmental remediation, CTeMs effectively capture and decompose pollutants, offering both separation and detoxification. In sensor technology, they have the potential to provide high sensitivity and selectivity, essential for accurate detection in medical and environmental applications. For energy storage, CTeMs may be promising in enhancing ion transport, flexibility, and mechanical stability, addressing key issues in battery and supercapacitor performance. Biomedical applications may benefit from the versality of CTeMs, potentially supporting advanced drug delivery systems and tissue engineering scaffolds. Despite their numerous advantages, challenges remain in the fabrication and scalability of CTeMs, requiring sophisticated techniques and meticulous optimization. Future research directions include the development of cost-effective production methods and the exploration of new materials to further enhance the capabilities of CTeMs. This review underscores the transformative potential of CTeMs across various applications and highlights the need for continued innovation to fully realize their benefits.

## 1. Introduction

Membrane technology revolutionized numerous fields including water purification, medical diagnostics, and energy storage [1,2,3,4,5]. Among various types of membranes, track-etched membranes (TeMs) gained prominence due to their well-defined pore structures, high uniformity, and excellent mechanical stability. TeMs are produced by irradiating polymer films with high-energy ions followed by chemical etching, resulting in precisely controlled pore sizes and geometries [6]. These unique characteristics make TeMs particularly attractive for applications requiring high selectivity and precise molecular separation [7,8].

Despite their advantages, traditional TeMs face limitations in terms of functionality and adaptability. They typically exhibit passive behavior, with limited capacity for specific interactions or catalytic activity. To overcome these challenges, researchers modified them through methods such as grafting or developed composite track-etched membranes (CTeMs) by integrating functional materials into the TeM matrix [9]. This hybrid approaches combine the structural benefits of TeMs with the enhanced properties of added functional phases, such as metal nanoparticles, metal oxides, and various nanostructures in the case of CTeMs. The unique properties inherent in CTeMs, resulting from their versatile structures comprising various promising materials in one single body, render them indispensable for tackling contemporary challenges [10]. Characterized by precisely controlled porous structures, these membranes offer unparalleled opportunities in applications spanning from water purification and environmental remediation to sensing technologies, energy storage, biomedical fields, and beyond [11,12]. The key significance of CTeMs lies in their capability to provide tailored functionalities, empowering scientists and engineers to intricately design materials with precision. Beyond their promising applications, from a scientific perspective, CTeMs serve as a versatile platform for unraveling fundamental principles in materials synthesis and exploring structure–property relationships [13].

Understanding the contemporary landscape of CTeMs necessitates a comprehensive exploration of the historical development, synthesis techniques, and properties of their precursor, TeMs. The origins of TeMs trace back to the mid-20th century, marked by groundbreaking works that laid the foundation for their development, such as seminal contributions in nuclear particle detection [14,15,16,17,18]. These early utilizations underscored the intrinsic value of TeMs in particle physics, providing an innovative means for detecting and analyzing nuclear particles [19].

The synthesis of TeMs involves the intricate creation of nanopores within a thin membrane material. This complex process employs two fundamental methods that induce latent tracks within the membrane’s cross-section through irradiation. The first method entails irradiating membranes with fragments generated from the fission of heavy nuclei, such as californium or uranium. However, this approach faces challenges, including contamination of the tracked membrane with radioactive byproducts and limitations in creating varied angle distributions of pore channels. Moreover, the diverse masses and energies of fragments result in tracks with distinct etching properties [6,20].

A more refined and versatile approach to track etching is based on the utilization of ion beams from accelerators [6,21,22]. This method became foundational for TeM synthesis, enabling precise control over pore size, density, and distribution. The procedure initiates with the irradiation of a suitable substrate material, often a polymer such as polyethylene terephthalate (PET) or polycarbonate (PC), with high-energy ions, typically heavy ions or swift heavy ions. As these ions traverse through the material, they displace atoms along their trajectory, creating latent tracks within the material. These latent tracks subsequently serve as templates for the controlled formation of nanopores [23]. Following irradiation, the material undergoes either chemical or physical etching, a pivotal step that reveals the latent tracks and results in the formation of well-defined nanopores.

This method presents a straightforward and scalable approach to producing TeMs with meticulously controlled nanopores. Researchers possess the ability to fine-tune nanopore characteristics, including size and distribution, by adjusting parameters such as ion energy, etching conditions, and substrate material. Remarkably, ion track etching stands out as a versatile and widely embraced technique for large-scale production of TeMs, contributing significantly to the synthesis landscape. This advanced method not only ensures precise control over the structural attributes of TeMs, but also offers scalability, paving the way for diverse applications across scientific disciplines [24]. The nuanced adjustments allowed by this technique empower researchers to tailor TeMs according to specific requirements, underscoring its importance in the synthesis of advanced functional materials.

The evolution of TeMs over subsequent decades underwent a nuanced refinement encompassing properties, performances, synthesis, and modification techniques, fostering advancements that transcend conventional boundaries [25,26]. This iterative process not only elevated the intrinsic attributes of TeMs, but also propelled the exploration of diverse methods aimed at imparting additional functionalities to these membranes. CTeMs are specialized porous membranes consisting of a TeM matrix that undergoes deliberate modification through the integration of additional materials, such as metal or metal oxide nanoparticles, thereby creating a multifunctional and tailored structure with enhanced properties for specific applications, ranging from environmental remediation to biomedical sensing and beyond [13,27,28].

The merits of TeMs, and by extension CTeMs, lie in their meticulously controlled porous structures, characterized by high permeability, outstanding uniformity, and well-defined pore size distribution. Furthermore, the straightforward fabrication process and the capability to customize membrane properties enhance the attractiveness of TeMs for diverse applications. The expansive utility of TeMs and CTeMs spans various scientific and technological domains. In the realm of water purification and remediation, the precision in controlling pore size enables selective filtration, facilitating the removal of contaminants such as heavy metal ions, organic pollutants, and dyes. Furthermore, CTeMs find relevance in catalytic applications, showcasing their potential in promoting chemical reactions efficiently. Moreover, their versatility extends to biomedical applications, sensing technologies, and beyond. Examples include advanced sensors for environmental monitoring, biomedical sensors for healthcare applications, and selective membranes for efficient separation processes in various industries. The adaptability of CTeMs across this diverse array of applications underscores their significance in advancing solutions to complex challenges in different scientific and technological fields.

While composite track-etched membranes (CTeMs) demonstrated significant promise, especially in environmental applications and catalysis, several challenges must be addressed to unlock their full potential. One key issue is the long-term stability and reusability of the embedded nanostructures, particularly under harsh operational conditions such as high pressure, extreme temperatures, or corrosive environments. Another challenge is the scalability of the membrane fabrication process. Although track etching techniques offer precision, they can be costly, time-consuming, and require sophisticated instrumentation, making large-scale production difficult. In terms of recent advancements, studies show remarkable progress. For instance, CTeMs embedded with silver [29], gold [30,31], and copper [32] nanoparticles significantly enhanced their catalytic efficiency in environmental remediation. Likewise, a study by Zhang et al. highlighted the potential of CTeMs in electrochemical biosensors, where an array of gold nanotubes (AuNTs) on a polycarbonate track-etched membrane enriched the surface for detecting bacterial DNA [33]. Despite these advancements, further research is needed to explore the full potential of CTeMs, particularly in biomedical and energy-related fields. Applications such as drug delivery, tissue engineering, and energy storage could greatly benefit from the unique properties of these membranes. Overcoming these challenges will require both advancements in synthesis techniques and a deeper understanding of CTeM behavior at the nanoscale.

This review aims to provide a comprehensive overview of the synthesis methods, functionalization strategies, and diverse applications of CTeMs. We will explore the pre-modification of TeMs through grafting techniques, various synthesis approaches for creating CTeMs, and their performance in different fields such as environmental remediation, sensors, biomedical science, and energy storage. Furthermore, we will discuss the challenges associated with the fabrication and application of CTeMs and propose future research directions to enhance their capabilities and commercial viability. In conclusion, the collective insights provided by this review underscore the remarkable versatility and potential impact of CTeMs across scientific, technological, and environmental landscapes, leaving open avenues for continued exploration and application in the future.

## 2. Modification of TeMs by Grafting Prior to the Production of CTeMs

Before presenting a detailed review of CTeMs, it is important to understand the foundational technique of grafting used to modify traditional TeMs. Grafting plays an essential role in many applications for several reasons. Although TeMs provide excellent physical structures, thanks to their well-defined and uniform pore sizes, they often lack the necessary chemical functionalities needed for more advanced uses. This limitation stems from the base polymers commonly used in TeMs, such as polycarbonate and polyethylene terephthalate, which are chemically inert and lack reactive groups or tunable properties such as responsiveness to external stimuli.

Grafting offers a versatile method to introduce a wide range of functional groups—such as carboxyl, amine, or hydroxyl groups—depending on the monomers employed. These functional groups can imbue the membrane with “smart” properties, such as responsiveness to temperature or pH, significantly expanding its utility in biomedical, environmental, and catalytic applications. Moreover, grafting with specific monomers can be tailored to improve compatibility with inorganic nanoparticles and biological systems, further enhancing the hybrid nature of composite membranes. This functionalization makes grafted TeMs highly suitable for applications in fields such as biomedical science, catalysis, sensing, and filtration [34]. The variety of monomers available for grafting adds flexibility and effectiveness, enabling the membranes to serve roles beyond their primary filtration functions.

The grafting process chemically modifies the membrane surface by attaching various functional groups, particularly polymers, enhancing the material’s properties and broadening its range of applications. This technique often acts as a precursor step in developing CTeMs, facilitating the integration of additional functional phases, such as metal or metal oxide nanoparticles. Through grafting, researchers can greatly enhance the selectivity, sensitivity, and overall performance of TeMs, paving the way for the advanced functionalities exhibited by CTeMs. The upcoming section provides an overview of the grafting techniques applied to TeMs, serving as a foundation for the more detailed exploration of CTeMs and their versatile applications.

In recent years, the synthesis and application of grafted polymers gained considerable prominence, with graft copolymerization emerging as a promising and versatile technique for modifying base polymers. This technique involves introducing various molecular functionalities into their structures, combining base polymers with specific monomers containing appropriate functional groups. The result is synthetic copolymers or modified natural materials with tailor-made specifications for targeted applications. Grafting different monomers onto base polymers can be achieved through various techniques, including chemical, photochemical, and radiation or plasma-induced methods [35]. The choice of technique depends on the properties of the base polymer and the monomers involved, as well as the desired degree of grafting.

For example, when a semi-crystalline membrane such as polyvinylidene fluoride (PVDF) is irradiated by accelerated heavy ions, stable radicals are formed in the membrane. After specific etching times, some of these radicals remain on the nanopore walls along the tracks. Carbon-centered radicals and peroxy radicals, which result from exposure to air in the etched zones, can initiate grafting under suitable conditions. Grafting can occur on the nanopore walls of track-etched membranes via these residual radicals when vinyl monomers are present [36,37,38]. Alternatively, post-irradiation grafting may be performed after the monomers diffused into the nanochannels [39,40]. In some cases, thermal or photoinitiators are introduced into the nanochannels to initiate grafting [41,42]. Numerous studies focused on grafting various polymers onto the surface and inside the nanochannels of TeMs. Table 1 provides a summary of some grafted monomers and the main properties imparted to PET TeMs, indicating the functionalization possibilities.

Conventional free radical polymerization techniques are typically used during grafting in nanochannels. However, to achieve more controlled architectures, controlled free-radical polymerization (CRP) methods, such as atom transfer radical polymerization (ATRP) [45,54,60,61] and reversible addition–fragmentation chain transfer (RAFT) polymerization [37,62,63] are applied in nanochannel grafting. These CRP methods offer precise control over the degree of grafting and the lengths of the grafted chains inside the nanochannels without blocking the pores, allowing for further applications. Regardless of the grafting technique, whether conventional or CRP, the process of modifying nanochannels in track-etched membranes represents a crucial and promising technology for developing innovative membranes. Regarding CTeMs, pre-modifying the nanochannels and membrane surfaces through grafting enhances both the functionality and nanoparticle loading capacity. These grafting-induced improvements significantly contribute to the success of targeted applications.

For instance, in 2018, Korolkov et al. synthesized gold nanoparticles (Au NPs) within the nanochannels of poly(acrylic acid) (PAA) grafted onto PET TeMs. This was achieved through radiation-induced reduction in an alcoholic solution using both electron beams and gamma radiation, resulting in a material highly effective in reducing 4-nitrophenol [64]. Before irradiation, Au^3+^ ions were absorbed by the carboxylate groups of PAA grafts on PET TeMs. PAA was chosen for its reported suitability as a complexing agent for Au ions and stabilizer for Au NPs. The incorporation of PAA allowed the creation of membranes with 312 ± 5 nmol/cm^2^ of COOH groups, which were utilized for the complexation of Au^3+^. The study reported that PAA formed stable complexes with gold ions, leading to the coating of PET TeMs and concurrently preventing the aggregation of Au NPs during subsequent radiation treatment. The research indicated that PAA-grafted TeMs could serve as an effective support material for the radiation-induced synthesis of Au NPs with varied sizes, with the catalytic activity depending on the size of the Au NPs.

In a recent 2022 study conducted by Parmanbek et al., a controlled polymerization technique, reversible addition–fragmentation chain transfer (RAFT) polymerization, was integrated into the grafting process to synthesize a well-defined poly(2-(dimethylamino)ethyl methacrylate) (PDMAEMA) grafted track-etched membrane surface before the reduction in silver nanoparticles (Ag NPs) via a reducing agent, hydrazine hydrate. The interior of nanochannels and the surface of PET TeMs underwent comprehensive and homogeneous modification through the applied UV-initiated RAFT-mediated graft polymerization. These grafted and Ag NP-decorated membranes were effectively employed for the removal of arsenic (As^3+^) [65]. The grafting of PDMAEMA resulted in a functional precursor surface, enhancing the stability of the loaded nanoparticles and actively participating in the removal of arsenic, thereby improving overall performance. The reversible addition fragmentation chain transfer (RAFT) polymerization technique utilized during synthesis yielded a homogeneous and uniformly grafted surface, essential for obtaining reproducible results. Furthermore, this polymerization method prevented the blockage of nanochannels during grafting by controlling the molecular weights of the grafts, thereby increasing the quantity and accessibility of the functionalized surface area. SEM-EDX elemental mapping confirmed the homogeneity of grafting and Ag NP loading, demonstrating the presence of Ag NPs inside the nanochannels facilitated by the applied controlled polymerization technique. The application of the RAFT polymerization technique proved beneficial in terms of enhanced performance, which aligns with other reported data [34,37]. Under optimal conditions, the As^3+^ removal efficiency after 10 h was 56.0% and 85.6% for membranes solely grafted and both grafted and Ag NP-loaded, respectively, while that of the PET template was significantly lower. Therefore, As^3+^ removal was significantly enhanced by both PDMAEMA grafting and Ag NP loading.

In another study by the same group, the grafting of poly(1-vinyl-2-pyrrolidone) (PVP) onto nanoporous PET track-etched membranes using RAFT polymerization was investigated. This method allowed precise control over the degree of grafting and the lengths of the grafts within the nanochannels of PVP-grafted PET TeMs. The functionalized grafted membrane absorbed palladium ions, which were then reduced into palladium nanoparticles (Pd NPs) using various chemical agents such as ascorbic acid, sodium borohydride and plant extracts, or thermal reduction. The resulting composite porous membranes were used in the photocatalytic degradation of the antibiotic metronidazole [66]. The grafting process created a surface with a high sorption capacity for metronidazole and exerted a significant stabilizing effect on Pd NPs due to the functional PVP chains on the PET substrate. This study highlighted the smart combination of pre-modification of TEMs through grafting and subsequent loading of NPs, showcasing increased stability of loaded particles. The grafting methodology implemented prior to NP loading yields a fully functionalized surface that effectively contributes to the sorption and degradation processes, thereby enhancing overall performance.

Integrating grafting into the preparation of hybrid CTeMs containing NPs emerged as a relatively recent yet highly effective strategy. This technique provides a functionalized surface for the initial absorption of various metal ions, which are subsequently reduced into their nanostructured lower oxidation state counterparts, such as zero-valent metallic NPs, using various reduction methods such as chemical agents or thermal treatments. The promising outcomes observed in recent studies underscore the potential of this technique. As research in this field continues to evolve, it opens avenues for further exploration, providing opportunities to refine methodologies, expand applications, and uncover new insights. The dynamic nature of this area, considering both the versatility of polymers to be grafted and NPs to be loaded, suggests that future studies and enhancements will likely contribute to the ongoing advancement of CTeMs. Despite its potential, it is still early to consider this methodology a traditional method for synthesizing CTeMs compared to the other deposition methods mentioned in the following section, namely electrochemical, electroless, and radiation-induced deposition methods.

## 3. Synthesis of the Composite Track-Etched Membranes (CTeMs)

CTeMs became indispensable in a range of scientific disciplines, including physics, chemistry, environmental science, and sensor technology. These membranes combine the inherent physical, mechanical, and chemical properties of an inert polymer matrix with the high reactivity of micro- and nanostructures, which are deposited using various advanced methods. This combination results in highly robust, user-friendly, and cost-effective materials, emphasizing the importance of the ongoing development of new CTeMs. To synthesize CTeMs, several traditional deposition methods are employed, including electrochemical, electroless, and radiation-induced techniques. Each method offers unique advantages and faces specific limitations, contributing to the versatility and adaptability of CTeMs in different applications.

The synthesis of TeMs involves the intricate creation of nanopores within a thin membrane material. This complex process employs two fundamental methods that induce latent tracks within the membrane’s cross-section through irradiation [12]. The first method entails irradiating membranes with fragments generated from the fission of heavy nuclei, such as californium or uranium. However, this approach faces challenges, including contamination of the tracked membrane with radioactive byproducts and limitations in creating varied angle distributions of pore channels. Moreover, the diverse masses and energies of fragments result in tracks with distinct etching properties [6,20].

**Figure 1:**
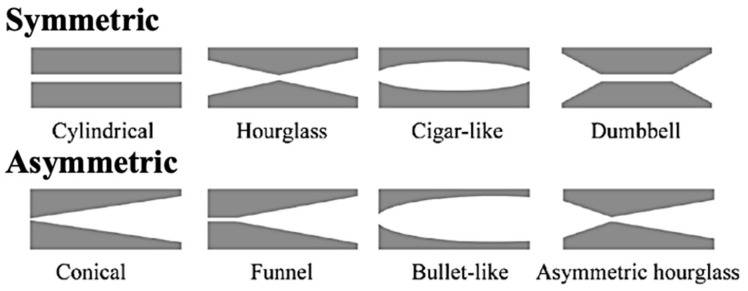
Symmetric and asymmetric polymeric nanochannels fabricated using the track etching technique (adapted with permission from ref. [72]. Copyright 2021 American Chemical Society).

A more refined and versatile approach to track etching is based on the utilization of ion beams from accelerators [6,21,22,67]. This method became foundational for TeM synthesis, enabling precise control over pore size, density, and distribution. The procedure initiates with the irradiation of a suitable substrate material, often a polymer such as PET [68,69] or PC, with high-energy ions, typically heavy ions or swift heavy ions. As these ions traverse through the material, they displace atoms along their trajectory, creating latent tracks within the material. These latent tracks subsequently serve as templates for the controlled formation of nanopores. After irradiation, the material undergoes chemical or physical etching, which reveals the latent tracks and results in the formation of well-defined nanopores [6,70,71]. By adjusting the etching conditions, both pore size and geometry can be controlled (Figure 1) [72]. A significant contribution to the understanding of track formation and chemical etching processes of TeMs was made by Professor Apel P. in numerous studies [73,74,75,76,77,78,79,80].

A crucial requirement for the etching solution is that the polymer should not swell in the selected etchant. This is particularly important for the polymer itself. Polymers with strongly hydrophilic properties tend to swell and undergo chemical degradation when exposed to an aqueous solution of an aggressive etchant, leading to poor track etching selectivity and gradual destruction of the polymeric template. As a result, all polymers used in industrial-scale production of track-etched membranes (TeMs) possess moderate hydrophobicity. For instance, the water contact angle for PET, polyethylene naphthalate, and PI is 73–75°, while for PC, it is around 80° [70]. The etching conditions for polymers used in commercial TeM production are listed in Table 2.

**Figure 2:**
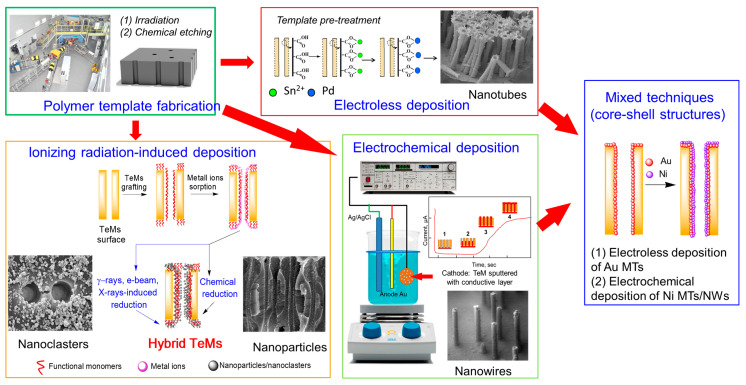
General synthesis routes for the preparation of CTeMs.

The choice and preparation of the polymer template are crucial for the successful implementation of the deposition process. The template must meet several specific requirements, such as chemical and physical stability, compatibility with processing conditions, and durability for repeated or long-term use. Specific needs may vary depending on the method used. For instance, in electrochemical deposition, a dielectric material should be used as the template. In all cases, the template material must remain chemically, physically, and thermally stable and inert during synthesis [81]. Additionally, the deposited material or solution must fully interact with the pore walls to achieve a uniform and thorough modification [82]. Various successful methods for synthesizing CTeMs, including electrochemical, electroless, and radiochemical deposition, were developed and are briefly illustrated in Figure 2. Each of these methods offers distinct advantages and limitations, which will be explored in detail in the following sections.

### 3.1. Electrochemical Deposition (ECD) for the Synthesis of Nanostructures in CTeMs

Electrochemical deposition (ECD) emerged as a prominent method for synthesizing CTeMs, allowing for precise control over the pore size and distribution within the membrane structure [83]. This electrochemical approach facilitates the creation of well-defined pores with tunable properties, making it a versatile and widely adopted technique in CTeM research. One of the earliest examples of synthesizing CTeMs through EDC was first proposed in 1969 during the deposition of silver in TeM channels. Subsequently, the technique for manufacturing cathodes of field electron emission based on ECD was patented by Spohr and later improved in 1984 by the group of Williams and Pshokvato [9].

For the electrochemical synthesis of individual wires with nano- and submicron diameters and lengths within the thickness of the TeM, it is necessary to establish a nucleation center for the galvanic precipitate directly in the membrane channels on one of its surfaces [84]. This can be achieved through vacuum spraying of a thin metal layer (50–100 nm). The electrochemical process of metal separation is localized at the electrode/solution boundary. Consequently, it is possible to use the matrix isolation (templating) approach for controlled nanoparticle growth. Precipitation with a controlled particle form factor is feasible by confining the electrochemical process flow zone to the walls of the porous matrix. A TeM with a sprayed metal layer is fixed in an electrolytic cell such that the sprayed layer maintains electrical contact with a metal ring under a negative potential (cathode). It is then pressed by a metal cylinder serving as an anode and placed in an electrochemical cell with the appropriate electrolyte. The anode is positioned parallel to the cathode in the solution. Cations diffuse to the cathode and are reduced when an electric field is applied, leading to an increase in nanoparticles inside the template pores [9].

Schönenberger and coauthors defined various successive stages of fibrous particle growth during ECD, observable in the current-time curve [81]. The initial section, corresponding to nucleation, shows a current maximum that can be described by the Scharifker and Hills model of nucleation under diffusion control. In the subsequent stage, fibrous nanoparticles grow within the template pores. As the metal/electrolyte growth front approaches the film’s outer surface, a slow increase in current density is observed, corresponding to the nanoparticle growth rate. Diffusion restrictions in the transfer of electroactive particles within the porous matrix result in an ohmic potential drop in the membrane’s long channels, which decreases as it approaches the surface. Under constant electrodeposition potential, this decrease in ohmic resistance leads to an increase in overvoltage and the growth rate of individual nanoparticles. The second section is determined by the particle growth rate and the porous membrane’s thickness. A sharp increase in current in the third section is associated with individual particles reaching the surface, accompanied by an increase in the metal phase’s surface area. In the final stage, the current growth slows or stops, indicating that the entire electrode surface is overgrown with metal.

Copper nanowires and nanotubes [27,85,86], silver [87,88], gold [89,90,91,92], platinum [93], nickel, cobalt, iron, bismuth, and cadmium, as well as various metal oxides, were obtained by electrochemical deposition methods in different polymer TeMs. Changes in ECD conditions also impact the content and crystal structure of the deposited nanostructures. For instance, when altering the composition of the copper nanotube deposition solution in PET TeM, it was found that using nitrate electrolytes and including glycine as a complexing agent results in the formation of oxide phases. Adding ethanol (10 g/L) as a surfactant to sulfate and nitrate electrolytes enables the production of monocomponent copper nanotubes with high crystallinity [94].

The ECD method is not limited to producing nanoparticles consisting of pure elements but also allows the synthesis of metal alloys with good stoichiometry control. By varying the potential in a solution containing different metal ions, nanoparticles with compositions such as Ag/Co [95], Co/Cu, Ni/Cu [96], and Fe/Cu [97] were synthesized. It was shown that the composition of Fe/Ni nanoparticles directly depends on the voltage magnitude and varies along the entire nanoparticle length [98]. Additionally, as the length of such nanotubes increases, their magnetic properties differ significantly in the parallel and perpendicular directions of the magnetic field, explained by the formation of a spiral-type anisotropy [99].

**Figure 3:**
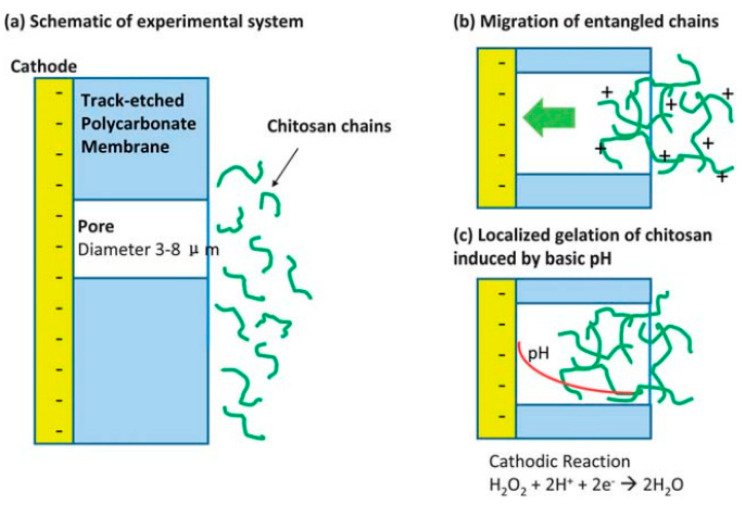
The scheme of chitosan ECD in the pores of PC TeM (adapted with permission from ref. [100]. Copyright 2005 Royal Society of Chemistry).

Beyond metal structures, electrochemical deposition can be used to deposit functional organic materials in TeM channels. For example, the stimulus-sensitive aminopolysaccharide chitosan was electrochemically deposited into PC TeM micropores (3–8 microns). The proposed mechanism for chitosan electrodeposition involves field-controlled migration of chitosan cation chains into the pores, followed by a sol-gel transition caused by localized neutralization of chitosan within the pore due to the high pH generated by cathodic electrolysis reactions (Figure 3) [100]. Recent work also demonstrated the possibility of obtaining metal-organic frameworks through the electrochemical oxidation of copper nanoparticles [101].

A series of research efforts explored combining chemical and electrochemical synthesis methods in TeM pores to produce core-shell Au@Ni nanotubes. CTeMs with chemically precipitated gold nanotubes were used as templates for further nickel electrochemical deposition. These nanotubes are widely used in sensing and catalysis and demonstrated high antibacterial activity against various bacteria types [102,103,104].

One of the main benefits of the ECD method is the ability to produce nanoparticles with high conductivity, as the process depends on electron transfer, which is most efficient along the conducting channel [105]. Structural analysis shows that nanoparticles obtained by this method are dense, continuous, and exhibit high crystallinity, unlike those produced by other methods such as chemical or vapor phase deposition [106,107]. Another significant benefit of ECD is the precise control it offers over the aspect ratio of metal nanoparticles, achieved by regulating the amount of charge passed through the system. This control is vital for practical applications, as the optical properties of nanostructures are highly dependent on their aspect ratios. Additionally, the ECD method allows for the coulometric control of the amount of embedded substance, facilitating the creation of nanostructures with a controlled geometric anisotropy factor by adjusting deposition modes and pore shapes. This method also enables nearly complete loading of the pore volume with the embedded material, and it can be performed at room temperature, thereby preventing thermal shrinkage and subsequent cracking of the samples.

### 3.2. Electroless Deposition (ED) Technique for the Synthesis of Nanostructures

Electroless deposition (ED) methods significantly contributed to the advancement of CTeMs synthesis. Unlike electrochemical deposition (ECD), which requires external current sources to supply electrons to reduce metal ions, electroless deposition relies on the catalytic oxidation of a reducing agent to provide the necessary electrons [108]. This method involves the controlled deposition of materials onto the membrane surface, which not only influences the structural characteristics of the membranes, but also introduces valuable chemical functionalities. By modifying surface properties through electroless deposition, TeMs can be tailored for specific applications.

The electroless deposition process includes stages of sensitization, activation, and precipitation. The first step (i.e., sensitization) consists of immersing the TeMs in an initial metal salt, such as tin salt in the presence of trifluoroacetic acid. During this stage, tin ions (Sn^2+^) ions bind onto the pore walls, leading to the sensitization process. As tin salts hydrolyze, they deposit poorly soluble hydrolysis products on the surface, forming a continuous layer several hundred nanometers thick. Therefore, the surface becomes hydrophilic and can bind ions of noble catalytically active metals. Once the membrane sensitized, the pore walls are activated by introducing an additional specific metal, such as a palladium (Pd) salt. This step aims to create metallic nuclei through a redox reaction between Sn^2+^ and Pd^2+^ ions. At the end of this step, the pore walls are decorated with Pd nuclei, which serve as seeds for the growth of the metallic layer. The final step, known as plating, involves reducing the metallic salt of interest using a reducing agent or process. This reduction results in the formation of nanocrystals on the pore wall and the membrane surface, ultimately yielding a fine metallic layer.

The deposition begins with the walls of the pores, differentiating it from ECD, where the size of metal nanoparticles (NPs) is controlled. In electroless deposition, the width of the NPs is determined by the width of the membrane track, while the length corresponds to the thickness of the applied matrix. Controlling the size of synthesized NPs in this method presents certain challenges. For instance, in the production of hollow nanotubes (NTs), the inner diameter is determined by the deposition time, while the outer diameter depends entirely on the size of the tracks in the template.

Early research by Ch.R. Martin on the chemical deposition of gold on polycarbonate (PC)-based polymer templates highlighted the potential of this method for developing composite track-etched membranes for various applications [109]. Gold deposition was one of the initial attempts used to control the geometry and dimension of asymmetric pores in track membranes. Over time, these composites found wide-ranging applications in sensors, selective filtration, biomedicine, and electronics [110,111,112]. One of the most widely used methods today involves synthesizing gold NTs into polymer templates using a sulfite precipitation solution. Significant contributions to the field were made by W. Ensinger and Dr. F. Muench, who investigated the nucleation and synthesis of NTs using different CTeM and electroless deposition methods [113,114,115]. A recent review by Prof. Muench summarized key advancements in electroless deposition, which may be of interest to readers [83]. Research on electroless template synthesis shows that using nitrogenous ligands such as ethylenediamine and pyridine improves the structure of silver nanotube (AgNT) composites [116]. The pH value of the solution also plays a critical role, affecting both the deposition rate and the structure of the resulting thin film. Low pH values and strong ethylenediamine ligands slow down the coating reaction, resulting in rough and porous films at low deposition rates, whereas smoother films are achieved at medium and high deposition rates. Depending on the desired physical properties of Ag nanostructures, either mode can be useful; for example, compact films for electrical conductivity or rough films for superhydrophobicity.

The efficiency of nanotube deposition is significantly influenced by the chemical oxidation of the membrane surface. For instance, a straightforward oxidative modification of PET TeM surfaces using hydrogen peroxide (H_2_O_2_) under UV irradiation was shown to enhance the structural integrity of deposited gold nanotubes. This modification not only improves the structural properties, but also significantly increases the reactivity of the composites in catalytic processes, such as the removal of p-nitrophenol (p-NP) [117,118]. Furthermore, this method proved effective in utilizing Cu@PET CTeMs as sorbents for arsenic (III) ions [119].

Temperature plays a crucial role in determining the structure of chemically precipitated copper nanotubes (NTs). Research demonstrated that lower temperatures result in the formation of copper crystallites with smaller dimensions, specifically 14.2 ± 1.5 nm at 2 °C compared to 19.6 ± 3.6 nm at room temperature [120]. A comparative study examining the ratio of [CH_2_O] to [Cu^2+^] in a formaldehyde-based copper precipitation solution identified the optimal conditions for synthesizing hollow copper NTs. The study found that using a low [Cu^2+^] content combined with a sixfold excess of the formaldehyde-reducing agent at temperatures not exceeding 13 °C yields the best results [121].

**Figure 4:**
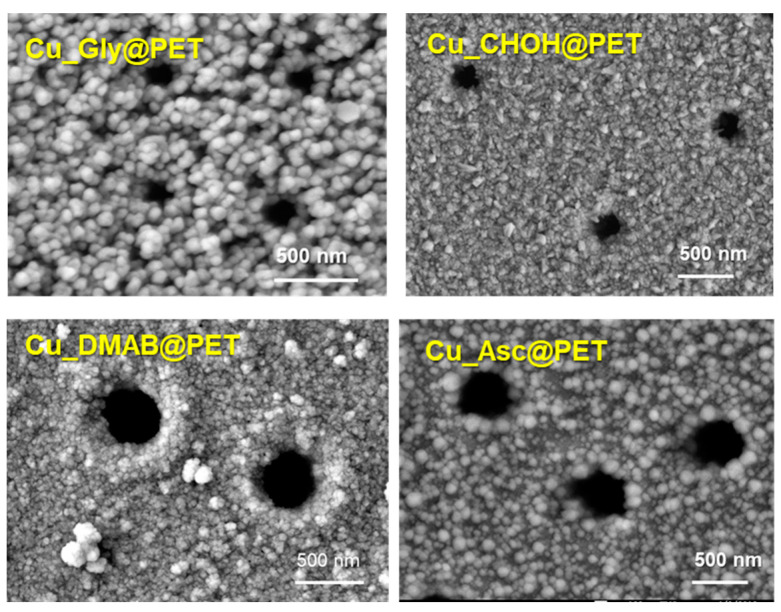
SEM images of the surface of CTeM with copper NTs obtained using various types of reducing agents (adapted with permission from ref. [122]. Copyright 2023 MDPI with license under CC BY 4.0).

A detailed investigation reported in [122] focused on the effect of the precipitation solution composition on the structure and properties of composite track membranes (TMs) based on copper metal. This study explored environmentally friendly reducing agents, such as ascorbic and glyoxylic acids and dimethylaminoborane (DMAB) (Figure 4). The research revealed that the presence of DMAB resulted in the formation of copper MTs containing the Cu_2_O oxide phase. In contrast, the use of ascorbic acid led to the deposition of copper in the form of nanoclusters across the membrane surface, forming thin-walled hollow structures.

Further advancements in the field include the development of multicomponent CTeMs using the galvanic displacement technique, proposed in 2015 [123]. This method successfully synthesized Ag–Pt NTs, where silver NTs served as the initial template. The mixed composition samples demonstrated high catalytic activity in the methanol oxidation reaction. In galvanic substitution, a precipitation solution containing a less noble metal reacts with a more noble metal, resulting in a spontaneous redox reaction. This method can synthesize various nanostructures suitable for applications in energy storage and catalysis. The reaction depends on factors such as metal ion concentration, pH, and temperature. Chemical deposition, typically restricted to a few monolayers without a catalyst, opens new opportunities by varying the composition of metal ions in solutions, thus developing functional materials.

**Figure 5:**
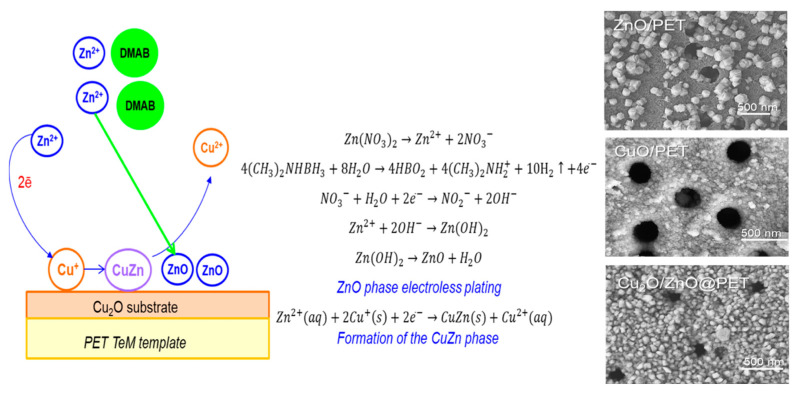
Scheme of Cu_2_O/ZnO@PET CTeM formation by galvanic substitution and SEM images of the studied composites (adapted with permission from ref. [124]. Copyright 2022 MDPI with license under CC BY 4.0).

Composites such as Cu_2_O/ZnO@PET were produced using galvanic substitution (Figure 5), revealing a CuZn substitution solid solution with a zhanhengite crystal structure [124]. Similarly, Cu/Ni_2_O_3_@PC composites were synthesized in two stages: depositing copper into a Ni_2_O_3_@PC template [125]. These samples, containing crystalline phases Cu Ni (97.3%) and CuO (2.7%), were tested in the photocatalytic decomposition of the antibiotic norfloxacin.

A key aspect of the electroless deposition (ED) process is that material deposition starts from the pore walls. This method offers several advantages, including the simplicity of the synthesis, the lack of need for specialized equipment, and the ability to perform the process using basic glassware. Another notable benefit compared to electrochemical deposition (ECD) is the ability to produce samples up to 20 × 30 cm in size. However, ED does have its limitations. Unlike ECD, where metal nanoparticle (NP) size can be controlled, in ED, the width of the nanowires (NWs) is fixed by the membrane track width, and the length of the resulting nanostructures is generally equivalent to the matrix thickness. Thus, controlling NP size is a complex task. For hollow nanotubes synthesized via ED, the inner diameter can be adjusted by the deposition time, while the outer diameter is dependent on the template track size.

### 3.3. Radiation-Induced Chemical Synthesis of Nanostructures in CTeMs

Radiation–chemical deposition techniques are a highly effective approach for synthesizing CTeMs, using ionizing radiation to induce controlled reactions within the membrane structure. Ionizing radiation, such as X-rays, gamma rays, or electron beams, is a potent tool in nanoparticle synthesis due to its ability to break chemical bonds and initiate a complex cascade of radiolytic reactions. When precursor materials are exposed to ionizing radiation in aqueous solution, the high-energy radiation imparts sufficient energy to atoms or molecules, causing the ejection of electrons and forming highly reactive species such as free radicals, cations, and anions. These reactive species play a pivotal role in the subsequent nucleation and growth of nanoparticles.

The radiolysis process begins with the ionization and excitation of molecules, mainly solvent molecules. This leads to the disruption of chemical bonds, often through homolytic cleavage, resulting in solvated electrons and free radicals. These initial species can recombine to produce molecular products. In aqueous environments, radiolysis generates specific species such as H_3_O^+^, H•, OH•, H_2_, and H_2_O_2_. Solvated electrons and H• atoms, in particular, act as potent reducing agents at ambient temperatures, making them highly reactive in initiating chemical reactions [126,127].

In the context of metallic nanostructure synthesis, radiolysis is a well-established mechanism where ionizing radiation reduces metal ions in the presence of a stabilizing ligand, eliminating the need for an external reducing agent. This process offers several advantages: it avoids contamination of metal sols by additives and ensures uniform distribution of reducing radicals throughout the solution [127]. The irradiated solvent itself acts as the reductant. However, it is crucial to mitigate oxidative radiolysis products, such as OH• radicals, which can counterbalance the reduction process of the metal ions. Strategies to scavenge OH• radicals include introducing molecules that generate inert radicals toward the metal particles or adding primary or secondary alcohols or formate ions, which generate additional reducing species.

**Figure 6:**
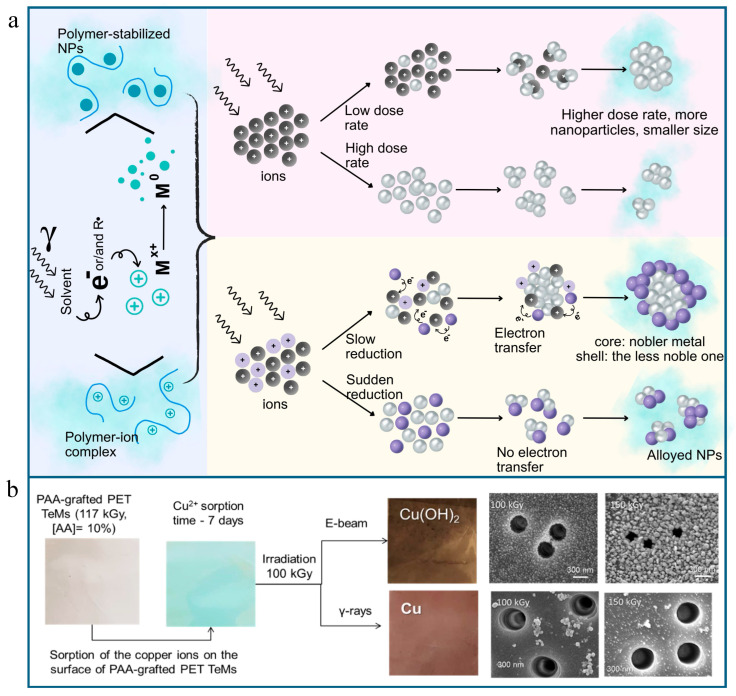
(**a**) Diagram showing the effects of different radiation dose rates on metal nanoparticle size (top panel). Diagram illustrating how the reduction rate affects the synthesis of bimetallic nanoparticles (bottom panel). Schematic representation of metal ion reduction in solution through ionizing radiation in the presence of a stabilizer (left panel). The blue cloudy shell around the ions or nanoparticles represents the capping/stabilizing organic phase, such as grafted polymer chains in a functionalized TeM. (**b**) Production methodology including grafting, sorption, and radiolysis for the synthesis of copper nanostructure-containing CTeMs using e-beam and gamma rays. The digital pictures and SEM images of the composite membranes are shown on the right ((**b**) is adapted with permission from ref. [128]. Copyright 2023 MDPI with license under CC BY 4.0).

The reactive-reducing species generated during radiolysis act as nucleation sites for the formation of nanoparticles, facilitating the aggregation of metal atoms or ions and promoting the initial nucleation step. As these nucleation sites accumulate atoms or ions, the nanoparticles grow, reaching their desired size and morphology. This process, depicted in Figure 6a, involves reducing a metal ion through radiolysis in the presence of a polymeric stabilizer, such as those grafted to the nanopore walls and surface of a TeM. The grafted polymers provide a high-capacity surface for metal ion absorption and stabilize the formed nanoparticles, preventing their aggregation during subsequent radiation treatment. Radiation can synthesize a wide array of metallic nanoparticles, including gold, platinum, ruthenium, silver, and palladium, as well as bi- and multi-metallic nanoparticles with alloyed or core/shell structures, such as AgAu, AgPd, AgPt, AgNi, and PdNi. It is also effective for fabricating oxide nanoparticles from metals such as chromium, iron, cobalt, rhenium, and uranium.

The radiation–chemical synthesis of CTeMs typically involves three stages. Initially, a membrane surface with absorption capacity, such as a polymer-grafted surface, is prepared, followed by the sorption of metal ions from saturated precursor solutions. The reaction mixture is then subjected to ionizing radiation, which can include electrons, X-rays, or gamma irradiation, leading to the reduction in the absorbed metal ions through radiolysis reactions. An example approach is illustrated in Figure 6b [128]. In this sample work, grafting a polymer such as poly(acrylic acid) (PAA) provides a functional surface with a high capacity to absorb metal ions, such as Cu^2+^. Following the irradiation of the Cu^2+^-sorbed membrane, copper nanostructure-containing composite membranes are obtained. The type of radiation used to reduce the copper ions affects the resultant nanostructures. For instance, gamma irradiation (^60^Co) yields predominantly metallic Cu phases, while electron beam (e-beam) irradiation results in mainly Cu(OH)_2_.

In Korolkov’s studies, the potential of using gamma radiation to reduce metal nanoclusters, specifically from the copper subgroup (Au, Cu), within a modified PET TeM matrix was demonstrated [64,129]. Copper nanoclusters of approximately 70 nm were synthesized both on the surface and within the channels of PET track membranes [129]. Further research by the same group included a comparative study of gold nanoparticle synthesis using an electron beam (in the dose range of 50–200 kGy) and ^60^Co (100 kGy). The gold nanoparticles obtained via the electron beam measured approximately 10–20 nm, while those synthesized using gamma radiation ranged from 15 to 40 nm. Both types of composites exhibited high catalytic efficiency in the reduction of *p*-nitrophenol (*p*-NP).

In general, the radiation–chemical deposition method offers several significant advantages. For instance, the additives introduced into the initial solution do not contaminate the resulting metal sols, thereby ensuring a high degree of purity and cleanliness. Additionally, during irradiation, reducing radicals are uniformly generated throughout the solution volume. However, one drawback of this method is the requirement for a source of ionizing radiation, as well as precise control over the dose rate, which plays a crucial role in synthesizing nanoparticles and nanoclusters with consistent dimensions.

### 3.4. Mixed Template Synthesis Techniques

**Figure 7:**
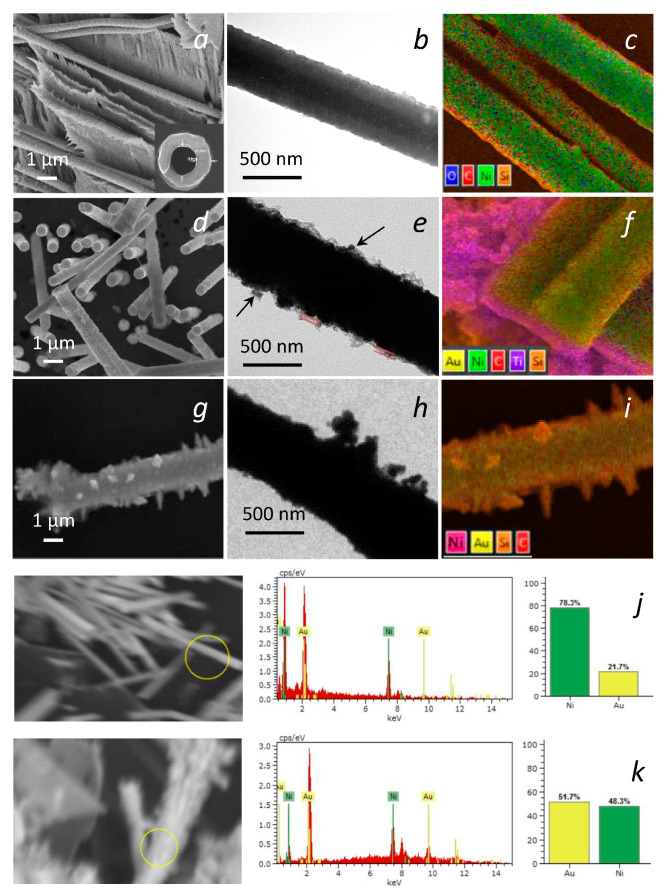
SEM images of Ar ion beam-etched PET TeMs with embedded Au microtubes (**a**), Ni dendrite structures on the unetched surface (**b**). SEM images (**c**), EDS spectra and mapping (**d**,**e**), and XRD patterns (**f**) of the core-shell Au/Ni microtubesand elemental composition (**j**). Ni@Au with gold needles: SEM (**g**), TEM (**h**), EDX-mapping (**i**), and elemental composition (**k**). Digital photographs and SEM images of the composite membranes are also shown on the right (adapted with permission from ref. [104] Copyright 2022 MDPI with license under CC BY 4.0).

Shumskaya et al. introduced a straightforward approach for synthesizing multicomponent composite track-etched membranes (CTeMs) containing embedded Ni/Au microtubes with a core-shell structure. These highly ordered Ni/Au core-shell microtube (MT) arrays were fabricated using a two-step template deposition method in the pores of PET TeMs. By varying the deposition techniques applied, different core-shell nanostructures could be achieved. For instance, Ni@Au core-shell nanotubes were synthesized through a two-step process: first, Ni nanotubes were grown inside the pores of the PET template, and then they were chemically functionalized with gold in an electroless wet chemical process, where the Ni nanotubes acted as the “shell” [130]. In another study, highly ordered Ni/Au core-shell microtube arrays with inner diameters of 110 ± 13 nm and wall thicknesses of 118 ± 5 nm were synthesized using a similar two-step template deposition method in the pores of track-etched membranes. Initially, Au microtubes were formed via electroless plating. In the second step, Ni microtubes were electrochemically deposited inside the gold microtubes, serving as the “shell” [102]. SEM images of these multicomponent CTeMs are shown in Figure 7.

To summarize, the literature extensively explored three advanced methods for synthesizing CTeMs: ECD, ED, and radiation–chemical synthesis. Each technique offers distinct advantages and was optimized to produce nanostructures with tailored properties for various applications. As we transition to the next section, we will explore the diverse applications of these CTeMs in fields such as environmental remediation, sensors, filtration, biomedicine, and electronics, underscoring the practical significance of these innovative synthesis methods.

Table 3 provide examples of CTeMs and their practical uses, particularly in areas such as environmental protection, separation processes, catalysis, biomedicine, and sensor technology.

## 4. Applications of Composite Track-Etched Membranes (CTeMs)

### 4.1. Environmental Applications of CTeMs

Ensuring water, soil, and air purity is critical for maintaining a healthy and safe society. As technology advances and populations grow, global challenges related to pollution continue to escalate. Addressing these challenges requires the development of sophisticated tools capable of detecting and mitigating both inorganic and organic contaminants. Recent strides in nanotechnology and materials science significantly enhanced our ability to detect, absorb, and catalytically degrade pollutants. TeMs, particularly composite ones integrated with metal-based nanoparticles (NPs), nanotubes (NTs), and nanowires (NWs), emerged as key players in catalysis, sensing, and filtration technologies. This section reviews recent advancements in utilizing these advanced materials for environmental applications.

#### 4.1.1. CTeMs as Catalysts in Water Purification

CTeMs offer numerous advantages as catalysts compared to nanoscale solid catalysts. Their ease of synthesis, availability, and low-cost polymer templates make them an economically attractive option for composite catalysts. What truly sets CTeMs apart is their high mechanical strength and chemical stability, ensuring reliability and durability as catalysts. A further advantage is their ease of operation: unlike nanoparticle powders or nanotubes that require time-consuming isolation procedures, CTeMs do not require additional activation or regeneration steps and can be easily removed from the reactor vessel after the process is complete.

CTeMs with catalytic properties present several benefits over traditional catalytic systems. Conventional catalysts, such as immobilized nanoparticles on inert carriers, often suffer from limited surface area and inefficient mass transfer. In contrast, CTeMs feature a highly organized, porous structure that increases surface area and enhances the distribution of catalytic material. The membrane matrix also offers mechanical stability, preventing catalyst agglomeration or deactivation during reactions. Furthermore, the well-defined pores in CTeMs facilitate the easy diffusion of reactants and products to and from catalytic sites, improving both reaction rates and selectivity. By embedding catalysts within the membrane, the process can be seamlessly integrated into filtration systems, combining catalysis with separation functions. This dual functionality is especially useful in applications such as water treatment, gas separation, and chemical synthesis, where simultaneous separation and reaction are required. Overall, CTeMs provide a more controlled, efficient, and durable platform for catalytic reactions than conventional sorbent carriers.

Catalytic reduction of organic compounds plays a pivotal role in both water purification systems and chemical manufacturing processes. In particular, the reduction of nitro-containing compounds, prevalent in industrial waste, garnered significant attention. Metal-based nanomaterials are widely recognized for their catalytic properties, underscoring the potential of metal-immobilized CTeMs as a burgeoning area of research. These membranes exhibit exceptional physical characteristics such as flexibility, mechanical robustness, inertness, and a high surface area, making them highly suitable for various environmental applications [120].

In exploring the applications of CTeMs in environmental remediation, their efficacy in the decomposition of organic dyes, antibiotics, inorganic compounds, and pesticides was extensively studied. A notable example of their catalytic activity involves the reduction of nitro-compounds, particularly *p*-nitrophenol (*p*-NP). For instance, Muench et al. (2011) introduced an innovative method involving the synthesis of silver-gold nanotubes (Ag-AuNTs) on polycarbonate (PC) TeMs for decomposing *p*-NP in the presence of sodium borohydride (NaBH_4_) [116]. The deposition of metals was achieved through electroless deposition, complemented by a coordination–chemical strategy to modulate silver’s activity using specific ligands such as ammonia (NH_3_), ethylene, and pyridine during plating. Subsequently, gold nanotubes (Au-NTs) were synthesized by treating Ag-CTeMs with tetrachloroauric acid. The study elucidated that ethylene proved most effective for the preparation of Ag-NTs. Reaction kinetics indicated that the reduction in *p*-NP followed Langmuir–Hinshelwood kinetics and exhibited pseudo-first-order behavior relative to p-NP concentration. This reaction mechanism was investigated using a flow mode system. Figure 6 illustrates the conversion of reactants, monitored by the decrease in absorbance at 400 nm corresponding to p-nitrophenolate, and the formation of products indicated by a peak at 300 nm. The study demonstrated that despite similar characteristics, the compact Ag-NTs and porous Au-NTs exhibited significantly different catalytic efficiencies, with the latter showing approximately seven times greater efficiency, underscoring the nuanced catalytic performance of different CTeM configurations in environmental applications.

**Figure 8:**
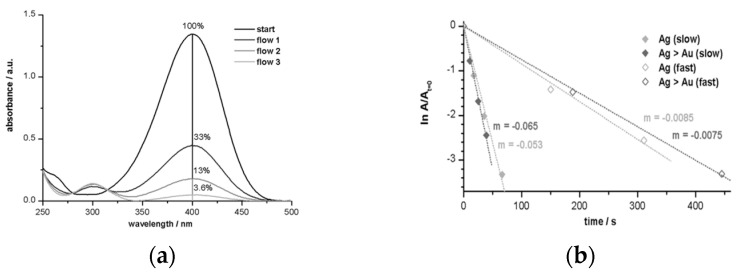
(**a**) UV spectra for reaction mixture, (**b**) calculated apparent rate constants for Ag and Au CTeMs (adapted with permission from ref. [116]. Copyright 1990 IOP Publishing Ltd.).

In 2014, Mashentseva’s research group published a study on the synthesis of gold/polyethylene terephthalate track-etched membranes (Au/PET TeMs) for the reduction in *p*-NP [155]. They introduced a novel approach for developing gold-based CTeMs, modifying the conventional method of electroless gold deposition with an Ag-based activation solution composition. The study compared Au-CTeMs produced using formaldehyde reductants (AS_II_) and an Ag-based solution (AS_I_). This modification allowed the production of two variations of PET TeMs fully coated with gold after reaction periods of 1, 5, and 24 h at 4 °C. Au/Ag/PET composites were synthesized by activating them for 3 min in an Ag-based solution, using potassium sodium tartrate as a reducing agent. The findings reveal that deposition time significantly impacts the reaction rate constant for both sample types (Table 4). For instance, the conversion degree of *p*-NP for the Au/PET catalyst (with a deposition time of 1 h and formaldehyde reductant) changed from 92.5% to 86.4% between the first and fifth cycles. The most effective catalyst was found to be Ag/Au/PET TeM, with a reaction rate constant of 0.087 min^−1^, compared to 0.041 min^−1^ for the Au/PET TeM catalyst (Figure 8). This highlights the importance of the activation method in enhancing the catalytic properties of these composites.

Research was also conducted on silver-based nanocatalysts, given silver’s excellent catalytic properties, similar to gold. In 2015, Borgekov et al. reported on the use of Ag/PET CTeMs for the catalytic decomposition of *p*-NP [29]. Silver nanotubes (NTs) were produced through electroless deposition on PET TeMs (12 µm, 1 × 10^9^ cm^−2^), with the plating method modified by the addition of sensitization in SnCl_2_ solution. In this study, p-NP was reduced to *p*-aminophenol (*p*-AP) in the presence of sodium borohydride (NaBH_4_), and the Ag/PET catalyst was rinsed in deionized water and reused for four cycles. The conversion degree of *p*-NP increased from 66.1% at 15 °C to 88.6% at 45 °C, with subsequent reactions performed at 40 °C. The economic and environmental significance of catalyst reusability garnered considerable attention recently. The reusability of Ag/PET composites was evaluated thrice at 40 °C, showing a gradual decrease in the steady rate, with observable surface damage on the CTeMs during the reaction [142]. Despite this, the studied composites demonstrated excellent reusability in the reduction of *p*-NP, as shown in Table 5.

**Figure 9:**
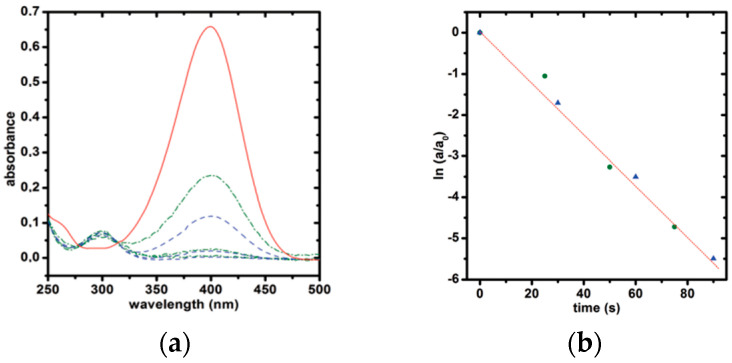
(**a**) The UV vis spectra of reduction of *p*-NP by Pd-based CTeMs synthesized by green approach, (**b**) graph of ln(a/a_0_) vs. time for the reduction in *p*-NP in the presence of Pd-based CTeMs (adapted with permission from ref. [156]. Copyright 1999 Royal Society of Chemistry).

Felix et al. (2016) conducted research on the synthesis and application of palladium (Pd) NTs for the reduction of *p*-NP [156]. The authors proposed a green method for preparing Pd NTs via electroless deposition on PC TeM, utilizing L(+)-glutamine and L(+)-ascorbic acid as environmentally friendly reductants. During the catalytic study of Pd-based CTeMs, excess NaBH_4_ was used to demonstrate the effectiveness of the prepared CTeMs. The findings indicate that the reaction followed pseudo-first-order kinetics, with a constant rate for the reduction of *p*-NP measured at 6.0 × 10^−2^ s^−1^. As illustrated in Figure 9b, the characteristic peak of the reaction product *p*-AP is at 295 nm, while the decreasing peak is at 400 nm. The red-colored line corresponds to the reference solution, and the three blue curves show the decreasing concentration of p-NP after each initial application step. Additionally, the three green curves (dashed lines) represent the reduction of *p*-NP after each stage of the application process over six months (Figure 9a).

In 2017, Korolkov et al. reported on the application of Au/PET CTeMs for the catalytic reduction in *p*-NP in the presence of NaBH_4_ [117]. During the electroless deposition of Au on pretreated PET TeM, the authors proposed oxidizing the PET TeMs in sodium peroxide (300 mM H_2_O_2_). The results show that oxidized Au/PET CTeMs (Au/PET-Ox) were 23% more catalytically active than etched template-based CTeMs (Au/PET-Etch). This increased activity was attributed to the enhanced hydrophilic properties of Au/PET-Ox. The study found that the constant rate for Au/PET-Ox CTeMs was 0.0466 ± 0.0031 min^−1^, whereas for Au/PET-Etch, it was 0.0358 ± 0.0023 min^−1^.

Mashentseva and coworkers, in 2019, presented research on the catalytic reduction in *p*-NP using copper (Cu) NTs-based composite catalysts in both flow and static modes [157]. The development of CTeM began with the preparation of a polymer template. PET TeMs (12 µm; 4 × 10^7^ ion cm^−2^) were chemically etched to obtain an average pore size of 395.20 ± 4.73 nm. Following sensitization in the SnCl_2_ solution and activation in the PdCl_2_ solution, electroless deposition was used to develop the CTeM. Activated PET TeMs were immersed in a deposition solution containing CuSO_4_·5H_2_O, KNaC_4_H_4_O_6_⋅4H_2_O, and NaOH at a low temperature (10 °C). Copper binding began with the addition of 0.13M formaldehyde for 40 min. The catalytic activity of copper nanotubes (CuNTs) on PET TeMs (CuNTs@PET TeM) was examined in both static and flow modes. The study of catalysts in flow mode evaluated the activity of pore spaces. The findings indicate that the highest constant rate was achieved in flow mode (Table 6). However, the reaction rate gradually decreased from 5.5 to 31 times after the second and third cycles, respectively. Initially, the average injection time for 10 mL of the reaction mixture through the CTM was 40–50 s, but this increased to 2–3 min in subsequent cycles due to pore blockage and *p*-NP adsorption on the inner nanowalls. During the first run in static mode, the duration was approximately 35 min, with an average increase of 5–10 min in subsequent trials; the sixth run lasted 60 min. These results demonstrate that while composite catalysts in flow mode offer a high rate of *p*-NP reduction, static mode achieves significant *p*-NP reduction at a lower reaction rate.

In 2020, the same research group investigated the application of Cu/CuO TMs for the catalytic degradation of various nitrophenols, including *p*-NP, *p*-NA, and *p*-nitro aldehyde (*p*-NBA) [32]. The authors suggested that the simplest and most cost-effective method for synthesizing CuO nanostructures is through the thermal annealing of copper. In this study, electroless deposition was performed as previously described [118]. Cu/PET TeMs were subjected to a thermal annealing process at temperatures ranging from 115 to 150 °C for different durations. The optimal conditions for thermal annealing were found to be 140 °C for 5 h. The prepared CTeMs were evaluated for their catalytic degradation of *p*-NP, *p*-NA, and *p*-NBA in the presence of NaBH_4_ as a reductant.

**Figure 10:**
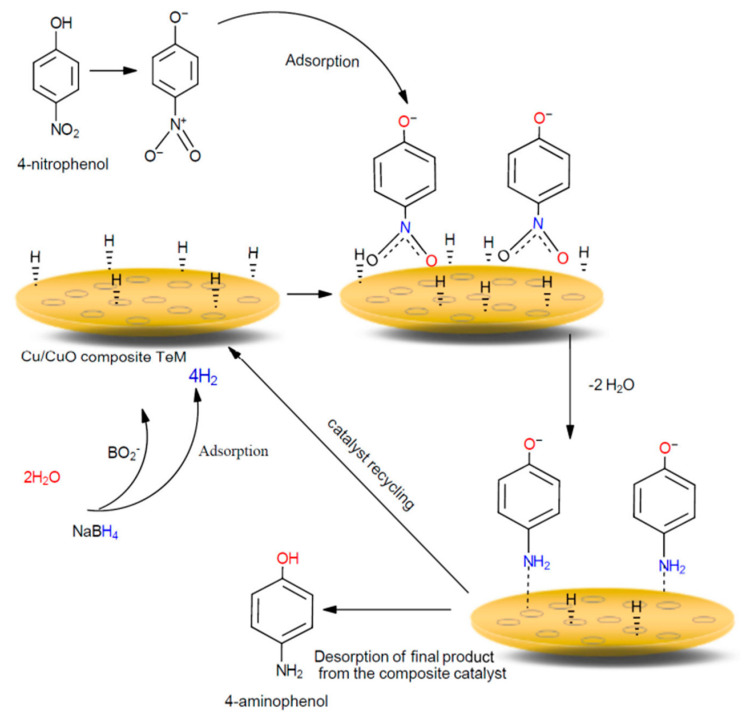
Mechanistic pathway of *p*-nitrophenol reduction on copper-based CTeM in the presence of NaBH_4_: Steps of the Langmuir–Hinshelwood mechanism, including adsorption, intermediate formation, and product desorption, involved in the catalytic reduction of *p*-nitrophenol on nanocatalyst surface (adapted with permission from ref. [32]. Copyright 2020 MDPI with license under CC BY 4.0).

Figure 10 illustrates the reduction in *p*-NP on copper-based CTeMs. The initial phase involves the adsorption of borohydride and nitrophenolate ions from an aqueous solution onto the surface of the nanocatalyst [158]. The subsequent stage consists of three steps: firstly, the equilibrated adsorption of 4-nitrophenolate occurs, resulting in the formation of p-aminophenol through the addition of hydrogen species and the creation of the p-hydroxyl aminophenol intermediate [159]. Secondly, two water molecules are eliminated from the nitro group. Finally, the *p*-aminophenol molecule detaches from the surface of the nanoparticle, making room for another catalytic cycle. The reaction mechanism follows the Langmuir–Hinshelwood mechanism [160], and the reduction in all nitroaromatic compounds proceeds with pseudo-first-order kinetics. Following this, the catalytic activity of the Cu/CuO CTeMs synthesized at 5 h of annealing time was investigated.

Table 7 illustrates the kinetic data for the hydrogenation of organic pollutants. The evaluation of the reduction in *p*-NA revealed that the catalytic performance of the annealed composite membranes (140 °C, 5 h) remained stable throughout five consecutive test cycles.

Platinum group metals are also utilized for developing CTeMs. Scheuerlein et al. (2020) reported research on the electroless deposition of iridium (Ir) on polycarbonate (PC) membrane for flow mode reduction of *p*-NP [139]. The application of Ir thin films or nanoparticles is promising due to their increased surface-to-volume ratio [161]. The researchers developed two Ir plating solutions suitable for creating Ir NP films. Complexing agents are commonly employed in electroless plating to maintain the stability of precursor ions and avoid homogeneous NP nucleation [162]. In the presence of a reducing agent, the complexes establish a metastable redox pair that exclusively reacts on catalytically active surfaces, subsequently advancing autocatalytically on the NP film. Trisodium citrate (Na_3_Citr) and ethylenediamine (EDA) were studied as potential stabilizers to maintain this metastable state throughout the Ir-plating reaction. Table 8 summarizes the content of both solutions. The reaction mechanism involves transition metal surfaces/NPs serving as catalysts, proceeding through a Langmuir–Hinshelwood reaction mechanism [163]. In flow catalysis, the Ir-coated membranes were manually subjected to a flow rate of 5 mL/min, with 5 mL of a freshly prepared reaction solution pumped through them. According to Lambert–Beer’s law, the concentration of a substance can be determined by measuring its absorbance. In this case, absorbance at 400 nm was used to determine the converted compound. The citrate deposits showed a conversion of 75%, while the EDA deposits exhibited a conversion of 80%. Catalysis was subsequently carried out in two different setups: a flow reactor configuration and a static setup where the membrane was immersed in a stirred reaction solution. Both deposits exhibited a conversion rate of approximately 93%, indicating a high level of catalytic activity. The catalytic performance of both deposits was similar to the results obtained from the *p*-NP experiments. Cyclic experiments indicated no clear trend towards a decrease in *p*-NP conversion over eight cycles, suggesting both catalysts could be reused multiple times without significant loss or poisoning of the *p*-NP compound. Despite distinct morphological differences, both deposits exhibited similar catalytic activities. However, overall performance was lower in static catalysis compared to flow reactor setup, with approximately 40% of *p*-NP converted after 5 min in static mode, whereas 75% conversion was achieved within 2 min in flow catalysis. The authors suggested that poor diffusion of reactants in the pores was the cause, making flow mode preferable to static mode.

Bimetallic CTeMs also show promise in catalytic applications. Research on gold nanostructures is extensive due to their high conductivity, localized plasmon resonance, and biocompatibility [164]. Gold nanoparticles, with their high surface energy and expansive surface area, are crucial catalysts in numerous reactions, offering exceptional activity and selectivity [165]. Developing inexpensive nanocatalysts is a promising direction, with many studies focusing on methods to stabilize gold nanoparticles and anchor them onto different surfaces to enhance their catalytic efficiency and robustness [166]. In 2021, Shumskaya et al. developed a bimetallic nanocatalyst, Ni@Au NTs, on PET TeM for the decomposition of *p*-NP in the presence of NaBH_4_ [103]. Ni NTs were obtained in PET TeMs pores by electrochemical deposition, followed by Au array deposition through a wet chemical approach. The potential impact of the nickel core on catalytic activity was assessed using spectrophotometry. Samples included Ni NTs and NTs with a ‘nickel core-gold shell’ of different gold coating morphologies and varying amounts of gold. Ni@Au (0.005) NTs led to a reduction in the absorption peak, while Ni@Au (0.01) NTs resulted in almost complete disappearance after 10 min, indicating nearly complete conversion of p-NP to p-AP. The reaction’s performance was assessed over five cycles with Ni@Au (0.01) NTs, showing an initial minor decline in efficiency (78% and 65% reduction compared to the first cycle), decreasing to 48% during the fifth cycle. The highest rate constant was achieved by Ni@Au (0.01) NTs—1.7 × 10^−3^ s^−1^.

**Figure 11:**
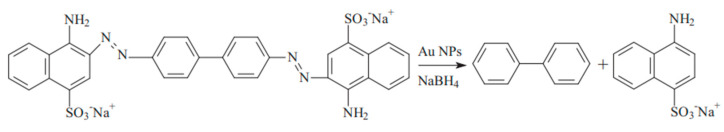
The mechanism of Congo red decomposition in the presence of NaBH_4_ (adapted with permission from ref. [30]. Copyright 2016 Elsevier).

Subair et al. (2016) studied the application of polydopamine-modified membranes with AuNPs for catalytic degradation of *p*-NP, methylene blue (MB) dye, and Congo red dye [30]. Dopamine was utilized to graft poly(ethyleneimine) (PEI) and create catalytically active track-etched PET membranes. Gold nanoparticles were produced through reduction in [AuCl_4_]^−^ ions fixed on dopamine (DOPA). Morphological analysis revealed gold nanoparticles on both the membrane’s surface and within the pore walls. The membranes were used in a flow-through membrane reactor for continuous flow catalysis and dye degradation. The significant decrease in *p*-NP and degradation of Congo red and methylene blue dyes at varying permeation rates indicates the potential effectiveness of these membranes. The *p*-NP to *p*-AP conversion rate reached 99% at a permeation rate of 40 L m^−2^ h^−1^. In contrast, reduction rates for Congo red (Figure 11) and methylene blue were 95% and 98% at 947 and 473 L m^−2^ h^−1^, respectively. The membrane exhibited consistent catalytic performance over an extended period, retaining over 99% activity after 11 cycles of dye degradation. These findings highlight the significant potential of membranes decorated with gold nanoparticles in catalytic processes and environmental applications.

The aforementioned studies on the decomposition of nitro compounds were systematically summarized in Table 9, which provides a comprehensive overview of the methodologies, catalysts, and kinetic data associated with each research. This table serves as a valuable resource for understanding the effectiveness of various composite track-etched membranes (CTeMs) in nitro compound reduction. With this foundation laid, the review will now proceed to explore the application of CTeMs in the decomposition of organic dyes, pharmaceuticals, pesticides, and inorganic compounds, as well as their use in catalytic reactions.

**Figure 12:**
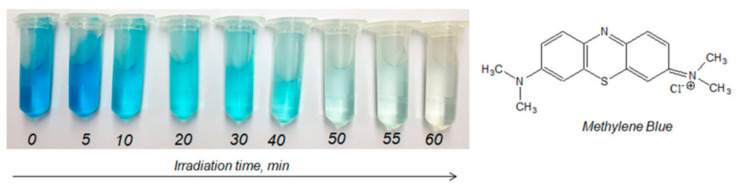
The change in color from blue to colorless was visually noted as a sign of MB (0.1 mg/L) degradation by the composite catalyst over various periods (adapted with permission from ref. [143] Copyright 2021 MDPI with license under CC BY 4.0).

Mashentseva et al. (2021) investigated the photocatalytic degradation of methylene blue (MB) using Ag microtubes (AgMTs) deposited on PET TeMs under visible light [143]. The study explored the influence of MB concentration, sample exposure time, and temperature on the catalytic performance of composite Ag/PET TeMs, along with the durability of their catalytic properties. After 60 min of exposure to visible light, a significant decrease in MB concentration was observed in the presence of Ag/PET composites (Figure 12). Conversely, identical experiments conducted without Ag/PET TeMs showed no substantial changes in absorbance even after 180 min of visible light exposure. The maximum degree of dye decomposition (D, %) was only 4.4%. The impact of initial MB concentration on degradation efficiency under visible light exposure was examined by varying MB solution concentrations from 0.1 to 5.0 mg/L. A significant reduction of over 85% was observed within 60 min at lower initial MB concentrations of 0.1 and 0.5 mg/L. In contrast, at higher concentrations of 1.0 and 5.0 mg/L, the reaction required 155 and 370 min of irradiation, respectively, to achieve over 90% MB decomposition. Analysis of experimental data suggested that increased MB concentration led to reduced decomposition efficiency due to the intensified color of concentrated solutions obstructing radiation penetration onto the catalyst surface [167]. Additionally, under identical experimental conditions, the proportion of hydroxyl radicals (•OH) to dye molecules decreased with increasing concentration [168]. The influence of temperature on degradation efficiency was investigated within the range of 17–58 °C, with an activation energy calculated at 20.6 kJ/mol. Experimental findings indicate that higher reaction temperatures enhanced MB decomposition by facilitating the movement of reactive radical species and the release of colorless decomposition by-products from the catalyst surface. According to the findings, the conversion of MB (1.0 mg/L) over 1 h reached 61.4%. The recyclability test demonstrated good reusability across 11 cycles, confirming sustained catalytic activity.

In the subsequent year, Altynbaeva et al. introduced a novel Cu_2_O/ZnO@PET TeM for the photocatalytic decomposition of carbendazim [124]. Carbendazim, a benzimidazole carbamate fungicide, poses environmental challenges due to soil and water contamination [169]. The composite TeM was fabricated via electroless deposition, where Cu_2_O/PET was initially used as a template and subsequently modified with Zn(NO_3_)_2_ and dimethylamine borane as a reductant. Mechanistic studies revealed that carbendazim photodegradation followed the Langmuir–Hinshelwood mechanism and a pseudo-first-order kinetic model. The activation energy Ea was determined to be 11.9 kJ/mol. The mixed composite Cu_2_O/ZnO@PET achieved over 93% carbendazim degradation after 140 min of irradiation, with stability testing confirming sustained activity across various temperatures over 10 runs. The decomposition rate constant was calculated as 1.76 × 10^−2^ min^−1^.

**Figure 13:**
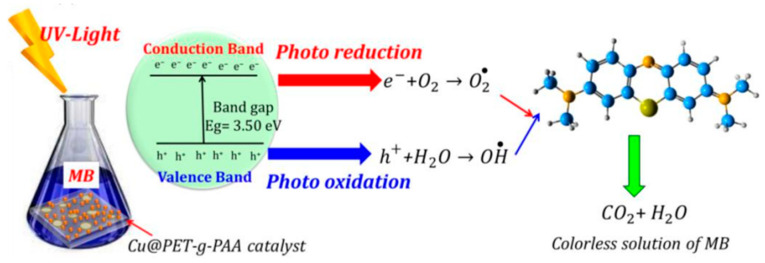
The mechanism for the photocatalytic decomposition of MB dye under UV irradiation in the presence of Cu@PET-*g*-PAA CTeMs (adapted with permission from ref. [128] Copyright 2023 MDPI with license under CC BY 4.0).

Recently, Parmanbek et al. (2023) investigated the catalytic properties of copper nanoclusters on PET TeMs [128]. Their study aimed to evaluate hybrid composites of PET TeMs integrated with copper nanoclusters (NCs) as catalysts for the photodegradation of methylene blue (MB) under UV light irradiation. Copper NCs were attached to polyacrylic acid (PAA)-modified TeMs (PET-*g*-PAA) using electron beam irradiation or γ-rays treatment to form the catalyst [83]. Radiation-induced grafting proved advantageous over traditional chemical processes, offering controlled material composition without requiring catalysts or initiators and operating at room temperature to reduce energy and chemical consumption [170,171]. The synthetic protocol included PET TeM pretreatment as described in [118], followed by the addition of an aqueous acrylic acid solution with CuSO_4_ as the metal precursor. Experimental results demonstrate that Cu(OH)_2_@PET-*g*-PAA composite TeMs exhibited superior catalytic activity in MB dye photodegradation. Under UV light exposure, the hybrid composite achieved 91.9% dye degradation, whereas Cu@PET-*g*-PAA samples achieved 83.9%. Figure 13 elucidates the charge carrier transfer mechanism during MB degradation using Cu@PET-*g*-PAA composite membranes, where PET-*g*-PAA acted as a support for Cu nanoclusters [172]. Photon energy absorption by these nanoclusters generated electrons and holes, facilitating electron transfer to the conduction band and hole formation in the valence band. Interaction between these bands and surface species generated superoxide and hydroxyl radicals, which effectively degraded MB into non-toxic compounds [173]. The porous nature and high surface area of Cu@PET-g-PAA composites provided abundant active sites, enhancing radical generation and photocatalytic activity [174].

**Figure 14:**
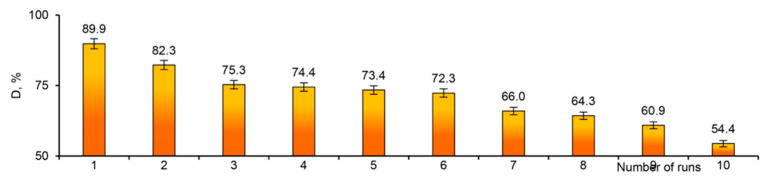
The reusability of the Pd_Asc@PVP-*g*-PET catalyst: change in the degradation degree (D, %) of MTZ in repeated use (adapted with permission from ref. [66] Copyright 2023 from the Royal Society of Chemistry).

Over recent years, nanoporous photocatalysts gained attention for their potential to decompose antibiotics in water sources due to environmental concerns from their use in veterinary and human medicine [175]. Parmanbek et al. introduced an environmentally friendly deposition of palladium (Pd) NPs on PVP-*g*-PET TeMs for the photocatalytic degradation of metronidazole [66]. Metronidazole (MTZ), an antibiotic, is known for its toxicity to humans and the environment [176]. The study involved modifying PET TeMs (12 µm, 4 × 10^7^ ion cm^−2^) via RAFT polymerization to control molecular weights and graft polymer chain architectures, addressing limitations of free radical polymerization techniques [171,177]. Utilizing a plant extract (*Betula Pendula Roth*) as a reducing agent, Pd NPs were prepared using NaBH_4_, thermal treatment, and ascorbic acid [178,179]. Experiments assessed reducing agent efficiency, pH levels, catalyst loadings, and MTZ concentrations in composite efficiency, where ascorbic acid-based catalysts demonstrated the highest efficiency, removing 89.86% MTZ at 30 mg/L. Optimal removal occurred at natural MTZ pH of 6.5, with efficiency decreasing as catalyst dosage and initial MTZ concentration increased. Reaction rate constants dropped from 0.0144 to 0.0096 min^−1^ as MTZ concentration increased from 20 to 50 mg/L, yet photocatalyst maintained high activity after 10 cycles (Figure 14).

In 2024, Mashentseva and co-workers investigated the implementation of mixed-composite TeMs for the photocatalytic decomposition of norfloxacin under UV light [125]. Authors utilized galvanic replacement to replace Ni_2_O_3_ microtubes (MTs) initially deposited via electroless methods in PC TeMs with NiCu MTs. Extensive research on both initial (Cu@PC and Ni_2_O_3_@PC) and resulting (Cu/Ni_2_O_3_@PC) composites emphasized their superior photocatalytic degradation efficiency for norfloxacin (NOR). Tests indicated 59.15% NOR removal within 180 min under UV light, with Cu/Ni_2_O_3_@PC adhering to a pseudo-first-order model and exhibiting a higher rate constant (k_a_) of 0.55 × 10^−2^ min^−1^. Reusability tests over four cycles confirmed sustained catalytic performance, highlighting the pivotal role of hydroxyl radicals (OH^−^), superoxide radicals (O^2−^), and holes (h^+^) in NOR degradation [180]. Quencher tests with isopropyl alcohol, ethanol, EDTA-Na_2_, and *p*-benzoquinone implicated holes as the primary reactive species, compared to O^2−^ and OH^−^ radicals, significantly decreasing photocatalytic efficiency upon EDTA-Na_2_ addition [181].

Literature also covered limited studies on TeMs for inorganic compound decomposition. In 2015, Borgekov et al. explored silver-based TeMs for catalytic hydrogen peroxide decomposition with NaBH_4_ [29]. Catalysis occurred between 25 and 45 °C, with reaction times noted post 50 mL H_2_O_2_ addition and oxygen bubble detection via a glass burette. High temperatures favored both catalytic systems, with extended reduction times at lower temperatures. Table 10 summarizes data over three cycles. The reaction rate of the second cycle in the hydrogen peroxide decomposition reaction decreased by a factor of 2.4. Furthermore, the H_2_O_2_ conversion dropped to 54.7% upon second reuse, indicating the removal of the smallest-diameter active Ag from the composite membrane surface. This removal likely occurred due to the vigorous release of oxygen during the initial testing cycle. To confirm the removal and aggregation of Ag nanoparticles after the first run, an XRD analysis was promptly conducted. According to the Scherrer equation, the size of Ag crystallites slightly increased, measuring 14.54 nm after the first run at 40 °C and 18.52 nm after the second run.

Another study on hydrogen peroxide decomposition employed Ag/PET CTeMs [182]. Researchers synthesized Ag/PET CTeMs with varying pore densities of 1 × 10^9^ and 4 × 10^7^ ions/cm^2^ using an electroless deposition method as described in [183]. Table 11 outlines the catalytic activities of different Ag/PET CTeMs, with oxygen volume quantified using a glass burette. Results indicate that the catalyst activity is directly influenced by deposition time. For composites synthesized in PET TMs with a pore density of 4 × 10^7^ ions/cm^2^, the rate constant of the H_2_O_2_ decomposition reaction exhibited linear growth with increasing silver deposition time. However, in samples with a pore density of 1 × 10^9^ ions/cm^2^ and a maximum deposition time of 300 min, the reaction rate constant decreased by over 38% compared to those prepared within 60 min. Therefore, 60 min was chosen as the optimal deposition time for both membranes. Catalytic studies were conducted within a temperature range of 25–45 °C. Activation energy values for Ag/PET TMs with pore densities of 1 × 10^9^ and 4 × 10^7^ ions/cm^2^ were determined to be 34.35 and 39.25 kJ/mol, respectively. Additionally, Ag/PET samples exhibited an activation energy of 43.17 kJ/mol. In conclusion, Ag/PET CTeMs with a pore density of 4 × 10^7^ ions/cm^2^ demonstrated superior catalytic activity.

In the same study [182], the authors demonstrated the effectiveness of CTeM-based catalysts when compared to a similar non-porous catalyst (Ag-coated PET film). They found that the catalysts based on TeMs (with a pore density of 4 × 10^7^ ions/cm^2^ and a microtube inner diameter of 408.4 nm) decomposed hydrogen peroxide at a rate three times higher than that of the non-porous samples.

In 2021, Altynbaeva et al. investigated the degradation of potassium hexacyanoferrate (III) using Cu-based CTeMs [121]. The synthesis of CTeM involved an electroless deposition technique, with Ag/PET CTeMs fabricated with varying pore densities such as 1 × 10^9^ and 4 × 10^7^ ions/cm^2^. The study highlighted the potential environmental hazards of potassium hexacyanoferrate and the essential role of Fe(II) in human and animal metabolism. The authors emphasized the diverse practical applications of the reduction reaction of Fe(III) to Fe(II), including in the purification of tin, copper extraction from molybdenum ore, production of wine products and citric acid, and as model systems for antioxidant activity studies and medical diagnostics for diabetes mellitus patients. The catalytic activity of Cu/PET CTeMs was investigated, with results showing that composites based on tubular copper microstructures, synthesized using a non-toxic reducing agent (ascorbic acid), maintained high catalytic activity over six test cycles, indicating their promise as catalysts.

In 2022, Cu@PET CTeMs were evaluated for the catalytic reduction of Cr(VI) ions from water source [140]. This study explored the characteristics of composite track-etched membranes composed of copper microtubes, produced using different combinations of deposition solutions and diverse reducing agents: formaldehyde (Cu_CHOH@PET), dimethylamine borane (Cu_DMAB@PET), and glyoxylic acid (Cu_Gly@PET). The catalytic efficiency of the prepared composites was assessed in the reduction in chromium(VI) ions, revealing that single-component composites achieved chromium ion removal efficiencies ranging from 95% to 97%. However, the incorporation of a copper(I) oxide phase in Cu_DMAB@PET composites significantly reduced their catalytic activity, resulting in only 41% removal of the contaminant under similar conditions. The degradation of Cr(VI) followed the Langmuir–Hinshelwood mechanism and a pseudo-first-order kinetic model. The rate constant (*k*) for Cu_DMAB@PET composites was calculated at 0.017 min^−1^, over 9 times lower than composites synthesized with glyoxylic acid (0.156 min^−1^) and more than 15 times lower than those with formaldehyde (0.249 min^−1^). Temperature effects on composite catalytic performance were studied in the range of 10–38 °C, with Cu_CHOH@PET samples demonstrating the lowest activation energy of 10.8 kJ/mol.

In conclusion, CTeMs exhibit strong catalytic activity for decomposing both inorganic compounds and organic pollutants. For specific inorganic pollutants, adjustments can be made to the thickness of the nanotubes, the ion fluence of the TeMs, and the synthesis methods. Table 12 provides data on the decomposition of these inorganic pollutants.

**Figure 15:**
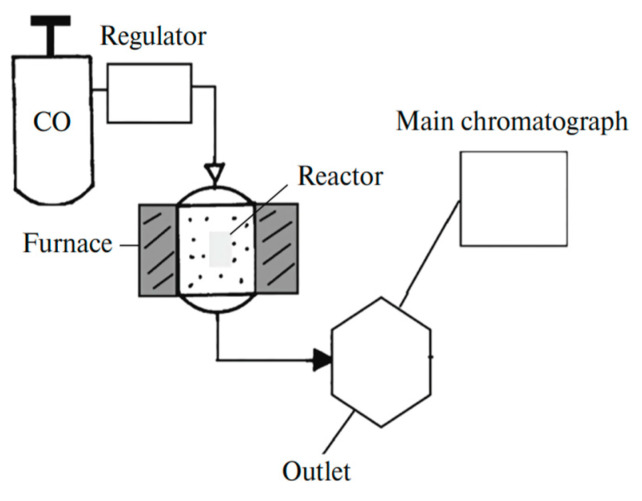
Schematic representation of installation (adapted with permission from ref [185]. Copyright 2021 Springer Nature).

Furthermore, copper-based CTeMs are applied in catalytic oxidation. Currently, commercially available Pt-Pd-Rh catalysts are employed for catalytic CO oxidation at temperatures above 400 °C [184]. Hence, there is significant interest in developing low-temperature oxidation catalysts. In 2021, Panov et al. reported on the application of copper nanowires (CuNWs) for CO oxidation using a flow-type installation schematically illustrated in Figure 15 [185]. The authors synthesized three types of copper CTeMs, varying in copper substrate shape: cylindrical, cone-shaped, and sandwich. Catalytic activity was assessed by measuring CO_2_ concentration in the gas mixture post-reaction. Cone-shaped CTeMs exhibited a 20% improvement in catalytic performance, while “sandwich” types demonstrated a 40% increase. Cylindrical nanowires achieved the best results, with catalytic activity reaching 70–80%. Post-catalysis, copper and cuprous oxides formed on the surface, along with carbon nanowires, precluding further application of CuNWs-based CTeMs.

#### 4.1.2. Application of CTeMs for the Heavy Metal Ions Sorption Removal

The next phase of scientific research focuses on water contaminant sorption mechanisms and describes various deposited metals. Various methods, including solvent extraction, resin-based ion exchange, and precipitation, were employed to eliminate ^137^Cs from aqueous waste solutions [186]. However, these techniques have limitations such as requiring large amounts of unwanted solvents and macrocyclic carriers, lacking selectivity, and producing significant secondary nuclear waste [187]. To address these challenges, the use of inorganic ion exchangers has the potential to mitigate some issues. Chaudhury et al. investigated the use of copper ferrocyanide nanocrystals-loaded track-etched membrane as a sorbent for Cs^+^ removal from neutral aqueous solutions [188]. The membrane was extensively characterized for Cs^+^ exchange kinetics and ion exchange capacity. Self-diffusion studies of Cs^+^ within this membrane indicated rapid Cs^+^ exchange kinetics. The presence of potassium in synthesized ferrocyanides facilitated faster cesium diffusion in the membrane compared to other cations. The membrane exhibited high efficiency in removing 99% of ^137^Cs activity, up to 3.8 × 10^5^ Bq, from a 15 mL water sample within 8 h. However, as solution volume increased, percentage activity removal decreased, even with extended equilibration times of 20 h. Additionally, the membrane effectively removed ^137^Cs from highly diluted solutions of 800 Bq/L.

Radionuclides are not the sole hazardous compounds present in industrial wastewater. For example, arsenic, classified as a class A human carcinogen, has a maximum allowable concentration of 10 μg/L in drinking water [189]. Phosphate (PO_4_^3−^) is a chemical analog of arsenate (As (V)) and is an essential nutrient but poses significant environmental hazards [190]. In 2020, Chaudhury et al. introduced an electro-membrane sorption approach for the selective sorption of As (III) and PO_4_^3−^ anions [191]. In contrast to capacitive deionization (CDI), direct interaction between the membrane (sorbent) and electrodes was obstructed by the aqueous solution, creating a barrier. By employing a two-compartment slow diffusion approach, researchers synthesized ferric oxyhydroxide nanoparticles within hydrophobic pores of a PC TeM (10 µm, 0.2 µm pore diameter). This separation of sorbent from electrode facilitated the use of various sorption materials, conductive or non-conductive, based on specific application requirements. Moreover, this method involved the targeted adsorption of desired species onto the membrane, followed by desorption from the membrane surface using an alkaline solution. Ferric oxyhydroxide (FeO) nanocomposite membranes (FeOm) were synthesized through diffusion-controlled growth of FeO nanoparticles within porous track-etched membranes, resulting in a sorbent with high capacity. The application of FeOm in the electro-enhanced sorption of phosphate revealed that an electric potential ranging from 5 to 15 V accelerated both adsorption and desorption processes. Desorption at pH levels around 10–11 was significantly lower than conventional pH levels of 13–14 for FeO regeneration, suggesting potential reductions in chemical consumption by 2 to 3 orders of magnitude.

**Figure 16:**
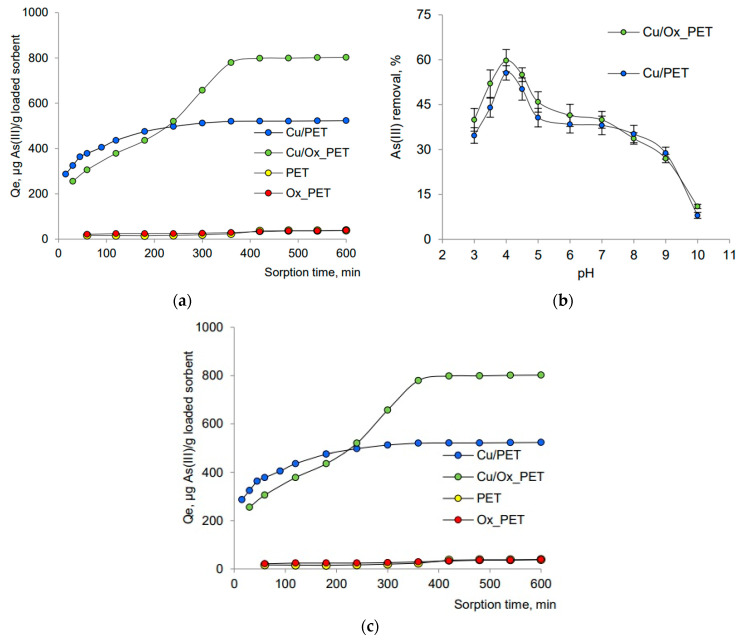
(**a**) Impact of contact time on the sorption of As (III) (50 ppm) by the composite TeMs, (**b**) dependence of As (III) removal (%) to pH during 420 min (**c**) (adapted with permission from ref. [119] Copyright 2021 MDPI with license under CC BY 4.0).

In 2021, Mashentseva and colleagues conducted further research into the selective sorption of As (III) [119]. Their study focused on producing and characterizing track-etched membranes (TeMs) integrated with copper microtubes via electroless deposition. They employed two types of TeMs: those made from etched-only polyethylene terephthalate (Cu/PET) and oxidized PET (Cu/Ox_PET) CTMs. The study also included a comparative analysis of arsenic (III) ion removal efficiency through batch adsorption experiments. Three kinetic models—Elovich, pseudo-first-order, and pseudo-second-order—were used to investigate the adsorption kinetics on composite TeMs. Figure 15 depicts the time-dependent adsorption of arsenic (III) by composite TeMs with deposited metallic copper microtubes. The impact of the PET template on sorption activity was evaluated using both pristine and oxidized PET species alongside both composite types. Figure 16a reveals that regardless of oxidation status, the PET template exhibits significantly low arsenic sorption capacities (Qe), with values of 33.2 µg As(III)/g for etched-only and 36.3 µg As(III)/g for oxidized PET templates. Oxidized PET as a template notably enhances equilibrium sorption capacity (Qe), reaching 802 µg As(III)/g for Cu/Ox_PET compared to 521 µg As(III)/g for unoxidized PET. The oxidation process extends the equilibrium sorption time slightly to 360 min, compared to 300 min for the pristine counterpart. However, this difference is negligible given the substantial equilibrium adsorption achieved. Investigation into pH impact on As (III) sorption demonstrated peak sorption capacity at pH 4 (Figure 16b), with equilibrium reached in approximately 6 h. The pseudo-second-order equation effectively described adsorption processes in both Cu/PET and Cu/Ox_PET composites. The study revealed a strong correlation between experimental data and the Freundlich isotherm, suggesting multilayer adsorption on varying energy surface levels. The use of an oxidized template enhanced As (III) removal efficiency, attributed to increased copper attachment, specific surface area, and surface porosity due to oxidation. Consequently, Cu/Ox_PET exhibited higher arsenic absorption due to these factors and smaller copper crystallite sizes observed with an oxidized template. The study also explored the influence of composite porosity on sorption activity. Figure 16c shows that using oxidized PET as a template significantly increases equilibrium sorption capacity (Qe) compared to etched-only PET. Specifically, the Qe for Cu/Ox_PET was 802 µg As(III)/g, while for unoxidized PET it was 521 µg As(III)/g. The limited effect of the PET template on adsorption suggests that this increase is primarily due to the larger amount of copper in the membrane and the higher specific surface area. Although the time to reach equilibrium sorption is slightly longer for oxidized PET (360 min) compared to the unoxidized version (300 min), this delay is minimal considering the substantial increase in equilibrium adsorption achieved.

**Figure 17:**
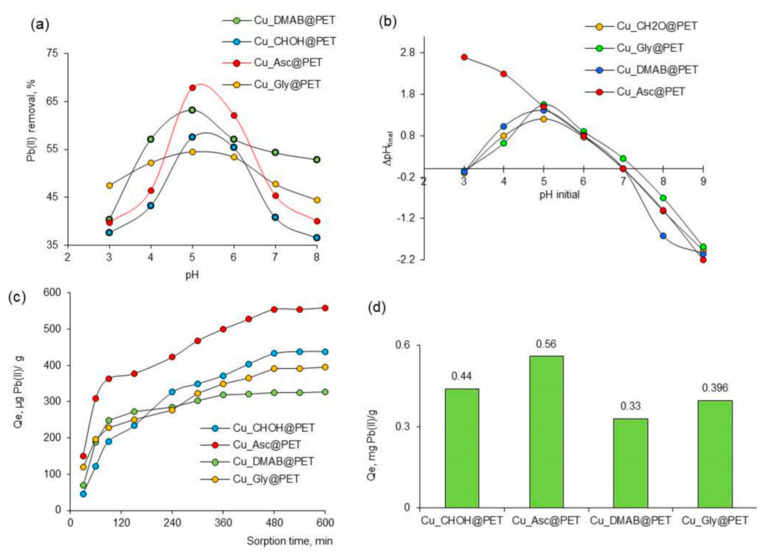
Sorption of Pb(II) as a function of solution pH (Pb(II) concentration: 50 ppm; contact time: 120 min) (**a**); pH point zero charge (pH_PZC_) plot (**b**); effect of contact time on the sorption of Pb(II) ions (**c**) and equilibrium sorption capacity (**d**) (adapted with permission from ref. [122] Copyright 2023 MDPI with license under CC BY 4.0).

Recently, Mashentseva and coworkers presented a study on Cu-based CTMs for Pb (II) sorption [122]. They synthesized CTMs modified with Cu microtubes via electroless deposition using environmentally friendly and non-toxic reducing agents such as ascorbic acid (Asc), glyoxylic acid (Gly), and dimethylamine borane (DMAB). pH significantly influenced Pb (II) adsorption, with optimal adsorption observed at pH 4–5 (Figure 17a). The pH limit of 8 was due to PET template decomposition in strongly basic solutions (pH > 9). In the pH range of 5–7, increased OH^−^ ion concentrations transformed Pb (II) ions to Pb(OH)_2_, hindering adsorption. The authors recommended investigating sorption below pH 6. pH at the point of zero charge (pH_PZC_) was crucial in determining neutral surface charge, impacting adsorption mechanisms and sorbent–adsorbate interactions (Figure 17b) [192]. Figure 17c illustrates time-dependent changes in copper microtube-based composite sorption capacity. Cu_DMAB@PET reached saturation in 360 min, while Cu_CHOH@PET, Cu_Asc@PET, and Cu_Gly@PET required approximately 480 min. Equilibrium sorption capacity (qe) from a 50 ppm Pb (II) solution varied by composite, with ascorbic acid exhibiting over 40% higher efficiency compared to DMAB (Figure 17d). Adsorption models—Langmuir, Freundlich, and Dubinin-Radushkevich isotherm — elucidated Pb (II) sorption behavior, highlighting chemical nature in Cu_Asc@PET and Cu_DMAB@PET and likely ion exchange in Cu_CHOH@PET and Cu_Gly@PET. The pseudo-second-order model best described kinetic adsorption across composites, with Elovich’s rate equation suitable for Cu_Gly@PET and Cu_CHOH@PET. These findings underscored the role of chemical interactions in determining rate-limiting steps in Pb(II) ion adsorption, with equilibrium typically achieved within 480 min, indicating adsorption was not solely controlled by intraparticle diffusion.

CTeMs demonstrate significant promise in environmental applications, fulfilling versatile roles such as filtration, absorption, and separation. Their precise pore size control, functional versatility, and robust mechanical properties enable effective removal of both organic and inorganic contaminants from water and air streams. CTeMs show considerable potential for use in industrial wastewater treatment, aquatic protection, and global environmental conservation efforts. In addition to their environmental applications, CTeMs are crucial in sensing technologies. They are adept at detecting a wide range of substances, including heavy metals such as Pb(II) [192], organic compounds such as acetaminophen [150] and methylene blue (MB) [193], polynitro compounds, and various other chemical contaminants, as well as biological structures such as DNA [194]. These applications underscore the diverse sensing capabilities of CTeMs. Further exploration into the utilization of CTeMs in sensor technologies, including detailed discussions on methodologies, performance metrics, and potential advancements, will be covered comprehensively in the following section of this review.

### 4.2. CTeMs in Sensor Technologies

Sensor technology became indispensable in modern society, driving advancements across diverse fields from healthcare to environmental monitoring and industrial automation. Sensors detect and respond to physical, chemical, or biological stimuli, converting them into measurable signals for analysis [195,196]. Despite their versatility, sensors encounter challenges such as limited sensitivity, selectivity issues, slow response times, and stability concerns under varying conditions [197,198,199]. These limitations are particularly critical in environmental monitoring, necessitating precise detection of pollutants, and in medical diagnostics, demanding accurate biomarker identification [12,67].

CTeMs have the potential to offer a transformative solution to these challenges in sensor technology. These membranes are distinguished by their high selectivity and sensitivity, attributes crucial for monitoring specific molecules or ions precisely. The ability to tailor CTeM surfaces with functional groups or incorporate nanoparticles and conductive polymers further enhances their specificity and sensitivity, enabling detection of low analyte concentrations. CTeMs exhibit rapid response times due to efficient mass transport properties and are characterized by robust polymer matrices that ensure durability and stable performance in diverse environments. Their versatility allows customization for various sensing applications, and scalable production potential promises cost-effective sensor solutions [67,200]. Integration of nanomaterials such as metallic nanoparticles and carbon-based nanomaterials further extends CTeM functionalities, enhancing electrical conductivity, catalytic activity, and surface area for improved signal transduction. These advancements underscore CTeMs’ potential in developing next-generation sensors that address critical challenges in healthcare, environmental monitoring, and industrial applications [12,67,200,201].

Bismuth (Bi), relatively non-toxic compared to mercury, emerged as a viable alternative to mercury electrodes for detecting heavy metals in wastewater and other applications [202]. Its strong affinity to form alloys and intermetallic phases with key heavy metals such as Pb, Cd, and Sn makes it particularly suitable for this purpose [203]. Scheuerlein et al. (2022) introduced the synthesis of Bi-coated PC TeMs (Bi-PC TeMs) for sensing Pb (II) cations [150]. The catalytic activity of bismuth was historically limited in decomposing many reducing agents used in electroless plating. However, recent breakthroughs show promise. A plating bath containing the highly stable EDTA complex of Bi (III) combined with borane dimethylamine (DMAB) as a reducer proved effective in achieving nanoscale Bi plating [149]. To validate this concept, the authors employed square-wave anodic stripping voltammetry (SWASV) for Pb detection using a specific coated membrane. Experimental conditions included a 0.1 M acetate buffer at pH 4.6, with gradual increases in Pb-acetate concentrations. The membrane, with a geometric area of 0.25 cm^2^, served as the working electrode in a traditional three-electrode setup, alongside an Ag/AgCl reference electrode and a glassy carbon counter electrode. SWASV involved a pre-concentration step at −1.2 V for 300 s, followed by stripping with a step size of 4 mV, pulse amplitude of 25 mV, and pulse frequency of 20 Hz. While further sensitivity enhancements are necessary for detecting Pb concentrations below regulatory limits in drinking water, initial trials demonstrate the potential of electroless-plated Bi for Pb detection. Optimizing sensor performance may involve strategies such as using larger pore diameters to facilitate quicker analyte diffusion within pores or adopting a flow-through configuration for detection.

**Figure 18:**
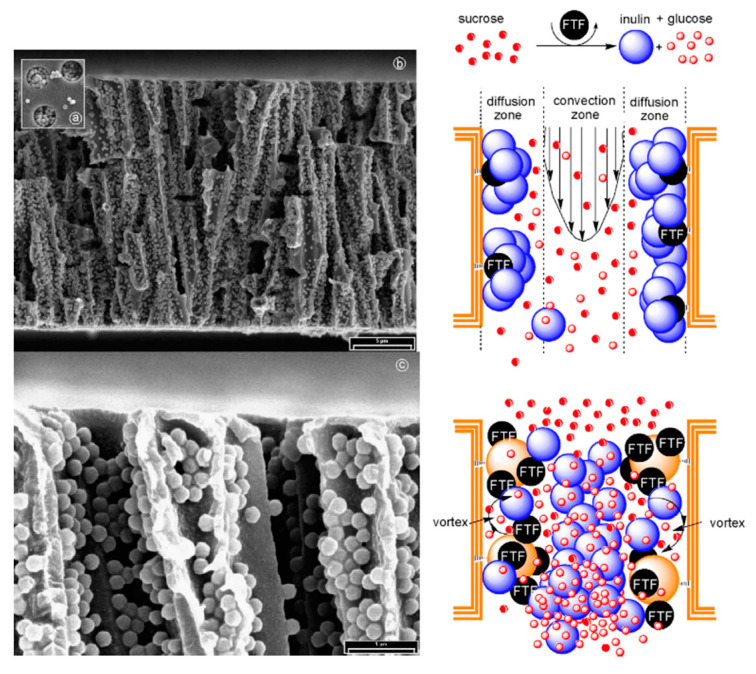
SEM of a nanoparticle composite membrane (nominal pore diameter 1000 nm; nanoparticle diameter 200–230 nm): filled pores before coupling reaction over-night (**a**), cross-section after coupling reaction and complete washing (**b**), cross-section detail demonstrating the distance between neighbored bound nanoparticles (**c**) and and depiction of the mass transfer and catalytic reaction behavior for the conventional enzyme membrane (**above**) and the nanoparticle composite enzyme membrane (**below**) (adapted with permission from ref. [204] Copyright 2006 Elsevier).

In 2006, Hicke et al. demonstrated enzyme–membrane reactors (EMRs) using amino-functionalized track-etched PET membranes with covalently immobilized fructosyltransferase (FTF) for continuous polymerization processes, particularly inulin synthesis [204]. Traditional EMRs faced issues of enzyme deactivation and pore blockage due to product aggregation, which were mitigated by incorporating reactive, spherical nanoparticles (200–230 nm) on membrane walls (Figure 18). These nanoparticles improved mass transfer, reducing pore blockage and enhancing reactor productivity. The development of nanoparticle composite membranes, with epoxy-reactive nanoparticles covalently immobilized on the pore walls, further optimized mass transfer and enzyme immobilization, highlighting their potential in biotechnology and chemical engineering applications.

The shape of the nanopores plays a crucial role in determining the performance and applications of these membranes. As it was mentioned in Section 3.1, TeMs can have cylindrical, conical, hourglass, or other geometrically defined pores (Figure 1). Each pore shape offers distinct advantages and impacts the membrane’s properties, such as flow dynamics, filtration efficiency, and sensitivity in sensing applications. Cylindrical pores, for example, provide uniform fluid flow and are often used in filtration where precise size exclusion is critical. Conical pores, on the other hand, can enhance particle capture efficiency and reduce clogging by facilitating easier passage of particles through the membrane. Hourglass-shaped pores are known for their ability to generate specific signal patterns in resistive pulse sensing, making them valuable in nanopore sensor technologies for detecting and characterizing nanoparticles [72].

In nanopore sensing, the geometry of the pore influences the magnitude and pattern of electrical signals generated during particle translocation, affecting the sensor’s sensitivity and resolution. Researchers and engineers tailor pore shapes to optimize performance based on specific application requirements, whether it is for biosensing, environmental monitoring, or industrial filtration. There is growing interest in using synthetic membrane nanopores as resistive pulse sensors for detecting biomedical analytes such as drugs, DNA, proteins, and viruses. Although this field is in its early stages, reproducibly fabricating artificial nanopore sensing elements is crucial for developing practical devices. In 2007, researchers evaluated conically shaped nanopores prepared by the track etch method in PET membranes, which have controllable large-diameter base openings and small-diameter tip openings [205]. The significance of controlling the tip diameter in resistive pulse sensing was demonstrated using nanopore sensors functionalized for protein analysis, specifically bovine serum albumin (BSA). Two types of conical PET nanopores with different tip diameters (17 nm and 27 nm) were utilized. These nanopores were initially lined with gold nanotubes and then coated with poly(ethylene glycol thiol) (PEG-S) to prevent nonspecific protein adsorption. It was observed that the current pulse magnitude (ΔI) plays a crucial role in detection sensitivity, where ΔI was significantly larger (80 ± 20 pA) for the nanopore sensor with the smaller 17 nm tip diameter compared to the sensor with the larger 27 nm tip diameter (ΔI = 35 ± 9 pA). This difference in ΔI was attributed to the more effective blockage of ion current by the BSA molecules as they translocate through the smaller nanopore tip, highlighting the importance of tip diameter control for enhancing detection sensitivity in resistive-pulse sensing applications. Using a new two-step etching process, they achieved good reproducibility and developed a mathematical model to predict tip diameters based on etching parameters, with predictions matching experimental results.

**Figure 19:**
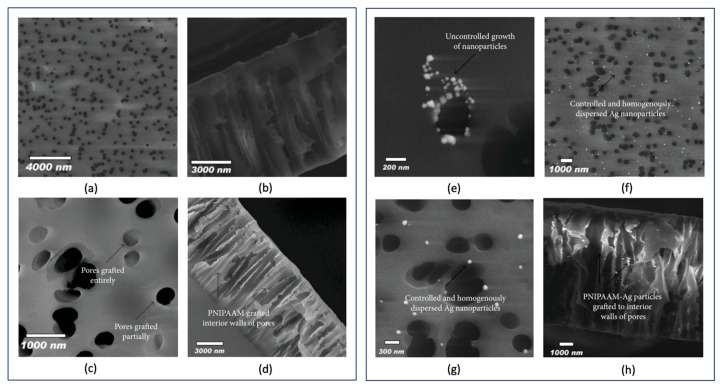
SEM images of the polycarbonate porous membranes without PNIPAM grafts (**a**,**b**), with PNIPAM grafts (**c**,**d**), without PNIPAM grafts after silver nanoparticles synthesized in situ (**e**), grafted with PNIPAM and after silver nanoparticles synthesized in situ (**f**–**h**) (adapted with permission from ref. [206]. Copyright 2014 Wiley with license under CC BY 3.0).

Stimuli-responsive or smart TeMs represent a cutting-edge class of materials with the unique ability to adapt and respond to various environmental triggers, such as pH, temperature, light, or chemical species. This responsiveness opens avenues for diverse applications ranging from selective filtration and controlled release systems to sophisticated biosensors and actuators. A study by Morones-Ramírez described the synthesis of intelligent optothermally responsive membranes by grafting polymer–metal nanoparticle nanocomposites onto polycarbonate TeMs [206]. Specifically, the synthesis method successfully incorporated PNIPAM-metal grafts into polycarbonate membranes, using PNIPAM as capping and stabilizing agents for silver (Figure 19). The nanoparticles serve as optothermal energy converters, enabling optical switching of the pores and allowing control of fluid flow. The study demonstrated that both polymer grafting and in situ synthesis of metallic particles are easily achievable, resulting in composite membranes that switch rapidly and reversibly using light and heat. These membranes exhibited controlled responses to temperature and light, affecting flow, and have potential applications as optically responsive valves for the delivery of bioactive agents, cell arrays, and advanced cell culture.

Surface-enhanced Raman spectroscopy (SERS) emerged as a powerful analytical technique for sensitive detection and identification of molecules at low concentrations. One of the critical components in SERS platforms is the substrate material, which enhances the Raman signals through plasmonic effects induced by nanostructured surfaces. TeMs, characterized by well-defined nanopores formed through controlled ion track etching in polymers, offer advantages such as high surface area-to-volume ratios and tunable pore geometries. These features enable effective adsorption and concentration of analytes, crucial for enhancing Raman signals. Functionalizing TeMs with noble metal nanoparticles to yield CTeMs further enhances their SERS performance by creating localized surface plasmon resonances that significantly amplify Raman scattering signals from molecules adsorbed on or within the membrane. In 2021, Ndilowe et al. immobilized silver nanoparticles (AgNPs) onto PET TeMs using diethylenetriamine (DETA) as a linker [207]. DETA formed an amide bond with PET following polyester ester bond scission, enabling covalent bonding of AgNPs to the modified PET membrane via silver-nitrogen bonds. The AgNP-coated PET membrane served as a SERS platform, detecting acetaminophen in water with strong Raman scattering intensity from adsorbed acetaminophen molecules on the AgNPs. Enhanced Raman scattering intensity on silver-coated track-etched PET membranes results from localized surface plasmons of silver nanoparticles, combining electromagnetic effects and charge transfer (chemical effects) from acetaminophen molecule absorption on silver nanoparticles.

**Figure 20:**
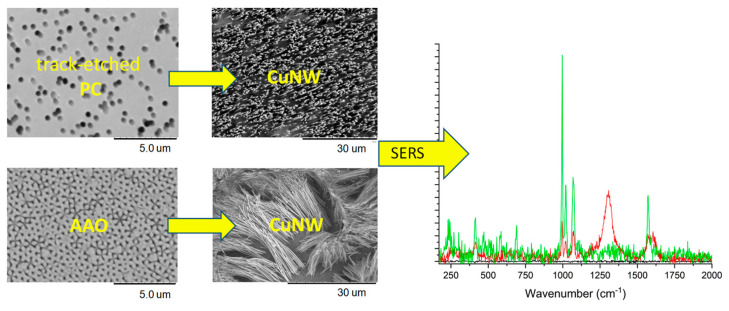
The preparation of arrays of copper ultramicrowires (CuUWs) by using porous membranes as templates track-etched polycarbonate (PC) and anodized aluminum oxide (AAO) for efficient substrates for surface enhanced Raman spectroscopy (SERS) (adapted with permission from ref. [208]. Copyright 2021 MDPI with license under CC BY 4.0).

A study by Longoni et al. focused on the electrochemical preparation of copper ultramicrowire arrays using porous membranes as templates for cost-effective and efficient substrates in SERS [208]. It compared anodized aluminum oxide and PC TeMs, and evaluated copper versus glassy carbon as electrode materials. Optimization of electrodeposition parameters through voltametric and potentiostatic tests led to the formation of copper ultramicrowire (CuUW) arrays, followed by template removal using NaOH for AAO and CH_2_Cl_2_ for PC. The study shows successful growth of CuUWs within polycarbonate membranes on both glassy carbon and copper electrodes, resulting in self-standing CuUW wires post-template etching. However, CuUW growth on anodized aluminum oxide (AAO) membranes was only feasible on copper substrates, yielding very high aspect ratio wires that are less mechanically robust and prone to collapsing after complete membrane etching (Figure 20). SERS spectra obtained from CuUW substrates using benzenethiol as a probe showed significant enhancement factors ranging from 10^3^ to 10^4^. Additionally, decorating CuUWs with silver nanostars demonstrated higher Raman enhancement, facilitating the formation of SERS-active hot spots at the bimetallic interface.

**Figure 21:**
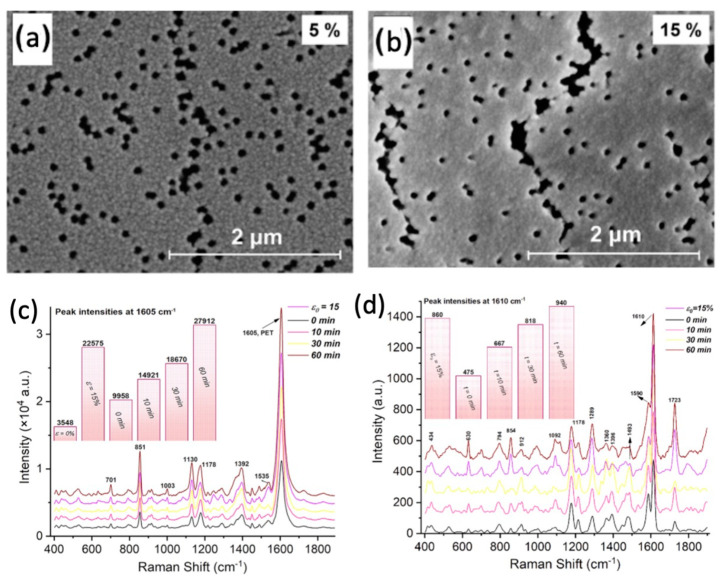
SEM image of metallized TMs surface elongated to deformation of 5% (**a**) and 15% (**b**), SERS spectra of malachite green molecules adsorbed on a surface metallized with a silver (**c**), and gold (**d**) (adapted with permission from ref. [209]. Copyright 2022 AIP Publishing).

Another interesting study by Kovalets et al. explores the intensified Raman scattering (RS) effect observed on microcracks in silver and gold metal depositions on uniaxially stretched polymer track-etched membranes [209]. Deforming these membranes, characterized by high surface density and small pore diameters, induces the formation of numerous microcracks within the metal coating. The efficiency of SERS on these synthesized metasurfaces was examined using malachite green as a model compound, demonstrating the ability to detect extremely low concentrations of the substance. The study also observed an increase in SERS signals with greater membrane deformation, further enhanced after unloading and relaxation (Figure 21). Tensile strain experiments and subsequent changes in electrical conductivity corroborated that SERS signals emanate from microcrack edges situated in close proximity. The research suggests using SERS to analyze the formation of micro- and nanocracks on metal coatings.

**Figure 22:**
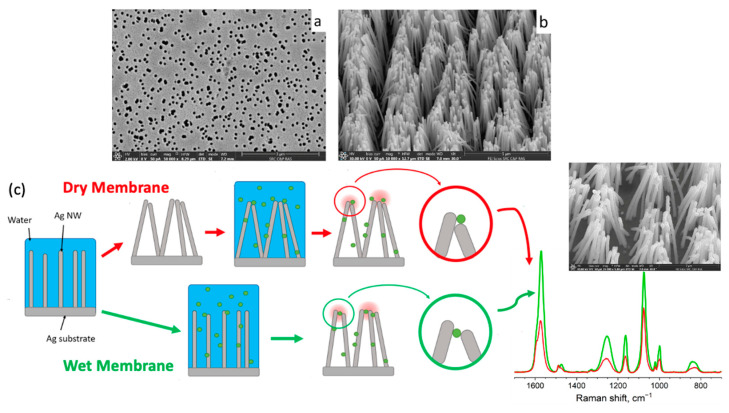
(**a**) SEM top view of the TeM, (**b**) Ag-NWs bundles array (Ag-NWs diameter of 100 nm and their length of 12 µm), and (**c**) mechanism of action for surface-enhanced Raman scattering (SERS) with metal nanowires (NWs) grown in pores of polymer TeMs and enhancement of Raman signal for 4-Mercaptophenylboronic acid (4-MPBA) adsorbed on the “wet” (green line) and “dry” (red line) substrates) (adapted with permission from ref. [87]. Copyright 2021 MDPI with license under CC BY 4.0).

A noteworthy study by Kozhina et al. presented a controlled synthesis of silver nanowire (Ag-NW) arrays with precise surface densities and diameters ranging from dozens to hundreds of nanometers, grown in pores of polymer track-etched membranes [87]. By adjusting deposition time, nanowire lengths varied up to micrometers. Specifically, this study focused on vertically grown Ag-NWs that naturally form self-assembled bundles, their configuration dependent on the nanowire aspect ratio (length to diameter). These bundles create “hot spots” at the nanometer-scale gaps between nanowire tips, where computer simulations predict significant enhancement in the electric field, amplifying Raman signals for analyte molecules situated within these gaps. Experimental validation using 4-Mercaptophenylboronic acid (4-MPBA) confirmed substantial enhancement of the Raman signal, particularly effective with nanowire lengths around 1.6 μm and diameters of approximately 100 nm. Moreover, the enhancement effect doubled when using a “wet” substrate immediately after polymer removal, facilitating optimal alignment of nanowire tips post-analyte exposure (Figure 22). This novel nanostructured SERS substrate holds promise for sensitive detection of analytes at extremely low concentrations.

In 2022, Kozhina and coworkers presented a method for forming magneto-optical one-dimensional (1-D) nanostructures through electrodeposition into track-etched membrane pores [210]. Two techniques were explored: synthesizing magnetic nanotubes (NTs) coated with silver and creating vertical-standing arrays of layered magneto-optical nanowires (NWs) alternating between nickel and silver. The study emphasized employing established template synthesis techniques to create magneto-optical arrays or powders of 1-D nanostructures incorporating plasmonic and magnetic metals. Nickel (Ni) was selected as the magnetic metal, and silver was utilized as the modifying plasmonic metal in array design. NTs powder synthesis employed PET track-etched membranes with specific characteristics: 12 µm thickness, 380 nm pore diameter, and 4 × 10^7^ cm^–2^ density. Ni@Ag NTs and layered Ag–Ni–Ag NWs were utilized as SERS-active substrates for detecting organic molecules, employing rhodamine 6G (R6G) as the test analyte to assess substrate sensitivity and reproducibility. The study found that layered Ag–Ni–Ag NWs exhibited a higher detection limit than Ni@Ag NTs, attributed to superior substrate nanostructure orientation and resulting plasmon resonance. SERS spectra analysis of R6G adsorbed on layered Ag–Ni–Ag NWs at a 10^−3^ M concentration.

**Figure 23:**
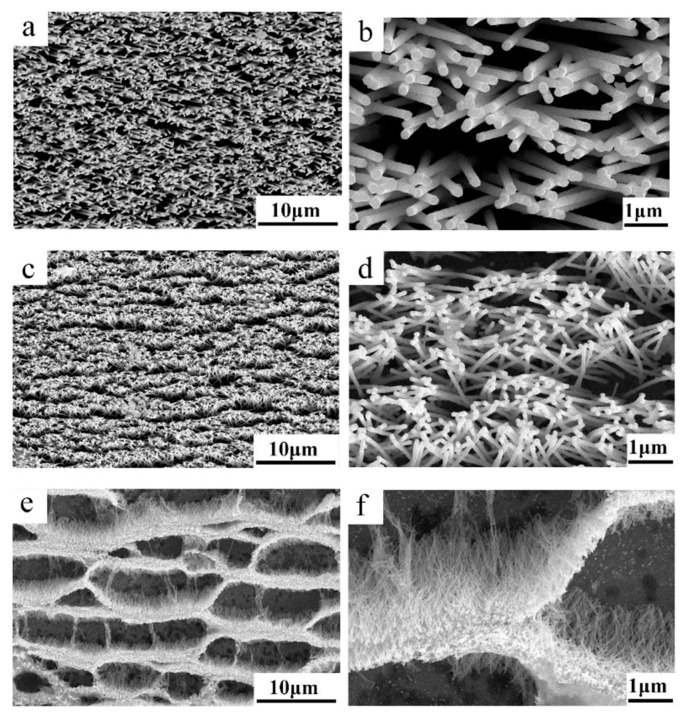
The SEM images illustrating metasurfaces featuring vertically standing nanowires (NWs) with varying diameters and surface pore densities: the substrate surface with NWs of 200 (**a**,**b**), 100 (**c**,**d**), and 60 nm (**e**,**f**) diameter and 10 µm length. (adapted with permission from ref. [137]. Copyright 2022 MDPI with license under CC BY 4.0).

Kozhina’s group later explored SERS mapping to address reproducibility issues observed in “homeopathic” concentrations of biorelevant molecules such as phthalocyanine (H2Pc*) [137]. They proposed SERS-mapping to analyze SERS parameter distributions for small (malachite green) and large (phthalocyanine, H2Pc*) molecules for metasurfaces featuring vertically standing nanowires (NWs) with varying diameters and surface pore densities (Figure 23). On substrates with 200 nm NWs, the NWs closely replicate the original track-etched membrane (TM) pore structure. Decreasing NW diameter to 100 nm results in more flexible NWs prone to agglomeration in characteristic strips, while 60 nm diameter NWs exhibit high flexibility, forming cellular structures due to capillary forces during drying, where adjacent NWs lean and form strips. These strips create localized electric fields at their tips, significantly enhancing SERS signals. Larger 200 nm NWs do not agglomerate tightly, forming autonomous bundles with fewer hot spots. The distribution of these hot spots crucially influences molecule adsorption and SERS activity on the metasurface. Optimal NW diameters for enhancing SERS signals are identified as 60 nm for small molecules (e.g., malachite green) and 100 nm for larger molecules (e.g., H2Pc*). This method emphasizes understanding the effective amplifying area and angular dependence between NW tips crucial for reproducible Raman spectra across substrates, presenting significant advancements in TEM-based sensor applications.

**Figure 24:**
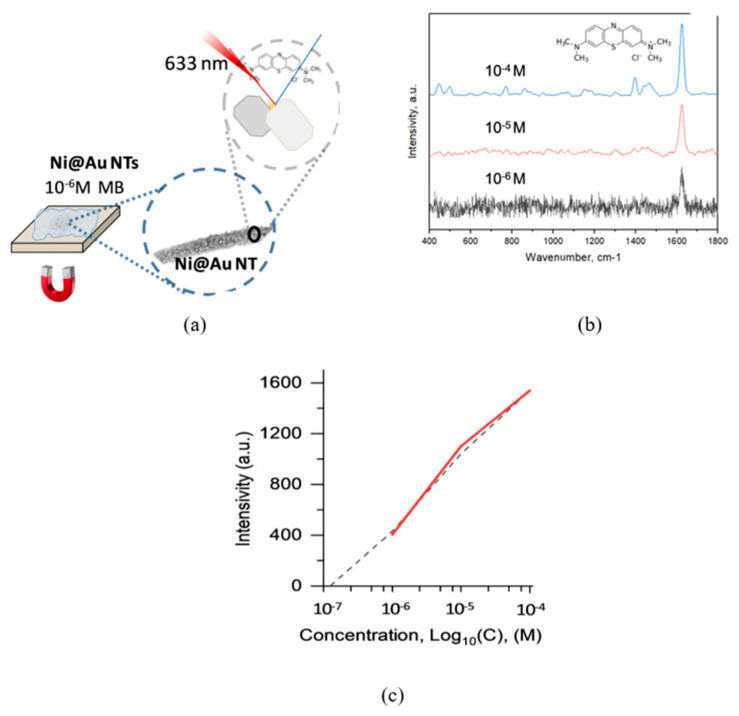
(**a**) Scheme of SERS experiment, (**b**) SERS spectra for various concentrations of MB, and (**c**) SERS intensity depending on the concentration of the 1624 cm^−1^ shift (adapted with permission from ref. [130]. Copyright 2021 Elsevier).

In 2021, Shumskaya et al. reported on the utilization of Ni@Au core-shell nanotubes (NTs) on PET TeMs for SERS detection of MB dye [130]. The synthesis of Ni@Au NTs, measuring 7.9 ± 0.2 µm in length and 470 ± 30 nm in diameter, was accomplished through a two-stage process. Initially, Ni nanotubes were grown within PET ion track templates, followed by the deposition of a nanostructured gold layer using electroless wet chemical methods. The SERS analysis revealed the plasmonic behavior of Ni@Au NTs, characterized by the formation of numerous “hot spots” due to the heterogeneous gold layer, enhancing the Raman signal. The enhancement factor (EF) was evaluated for MB concentrations up to 10^−6^ M (Figure 24a) [211]. To determine the limit of detection (LOD) of MB using SERS, the averaged spectra over five measurements exhibited prominent peaks at 1624 cm^−1^ (Figure 24b). The LOD of Ni@Au NTs for MB detection was approximately 1.3 × 10^−7^ M, indicating their potential for further research and SERS applications when compared to other plasmonically active metals [212] and nickel-containing structures [213].

In 2022, Shumskaya et al. presented research on the detection of polynitro compounds using Ni@Au NTs deposited on PET TeMs [104]. Ni@Au core-shell magneto-plasmonic nanotubes, 10 ± 0.2 µm in length, were synthesized via a two-step method. Nickel nanotubes were first grown within PET ion track templates, followed by electroless wet-chemical functionalization with a gold layer, either as a smooth coating or with nanoneedles. Despite functionalization, the magnetic core, nickel nanotubes, retained their original structural and magnetic properties. These nanotubes proved effective for SERS applications, demonstrating enhanced Raman signals for (NO_2_)_3_C_6_H_2_N(NO_2_)CH_3_ and C_6_H_3_N_3_O_8_ analytes at concentrations up to 10^−5^ M. To validate substance spectra reproducibility using SERS, simulated and experimentally obtained Raman spectra were compared, highlighting characteristic peak correspondence. Ni@Au core-shell magnetic nanotubes show significant potential for applications in chemo- and biosensors through active SERS.

**Figure 25:**
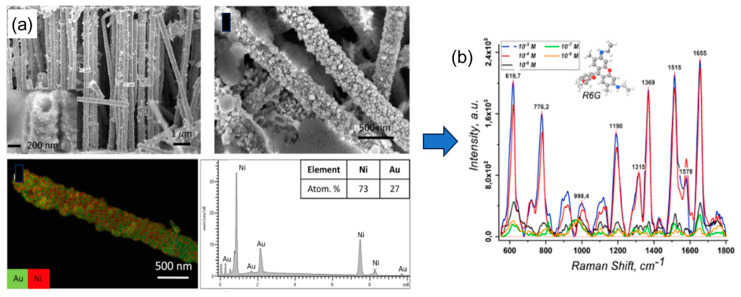
SEM images and SEM-EDX results of Ni-Au nanotubes (**a**) and their SERS spectra for different concentrations of R6G (**b**) (adapted with permission from ref. [214] Copyright 2022 Elsevier).

By selectively removing the polymer matrix of a CTeM, well-defined nanoporous architectures tailored for specific sensor requirements can be left behind. This capability allows for the creation of three-dimensional networks such as interconnected nanotubes or nanowires, which enhance the electrochemical and optical properties crucial for sensor functionality. In 2022, Korolkov et al. presented a dual approach involving the synthesis of substrates for SERS using magnetic nickel nanotubes (Ni NTs) and TeMs based on PET as templates [214]. Firstly, Ni NTs were coated with a uniform layer of Au via electroless deposition in alkali media, maintaining the magnetic properties of the nanotubes intact. Substrates featuring scattered Ni-Au NTs with a plasmon resonance peak at 530 nm demonstrated optimal efficiency for SERS applications using a 532 nm laser, illustrated by Rhodamine 6G spectra. SERS analysis of R6G on these substrates showed an average enhancement factor of 8 × 10^5^ across concentrations ranging from 10^−3^ to 10^−8^ M. Secondly, TeMs with specific pore characteristics were utilized as templates for the electrochemical deposition of Ni inside the pores. After dissolving the PET matrix with NaOH, the resulting Ni nanotubes were coated with a conductive layer of gold using magnetron sputtering. Subsequent electroless deposition of gold on these Ni nanotubes was achieved using a solution of Na_3_[Au(SO_3_)_2_], resulting in nanostructures in the form of nanotubes. A linear relationship was found in the logarithmic concentration range of 10^−6^–10^−8^ with R^2^ = 0.994, 0.996, 0.991, respectively. The proposed method of modifying magnetic nickel nanotubes (Ni NTs) with a uniform layer of gold (Au) through electroless deposition in alkali media significantly enhances the enhancement factor (EF) compared to previously published studies [103,215] that used nanotubes obtained by template synthesis (Figure 25). The entire process ensured open pores in the matrix, crucial for the formation of functional nanostructures. These methods demonstrated effective strategies for fabricating advanced SERS substrates and nanostructures with potential applications in sensitive analytical and sensor technologies.

**Figure 26:**
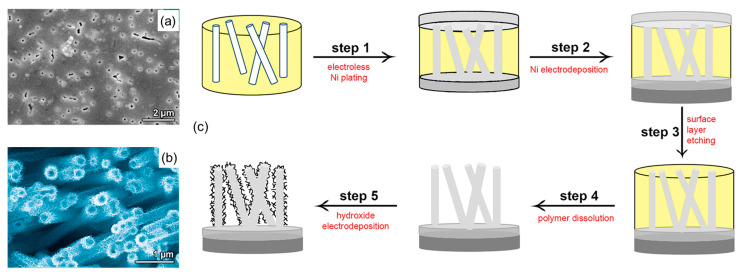
Top view of the NTNW before surface etching (**a**), SEM top view image of NiCo-LDH@Ni-NTNWs after 120 sec of hydroxide electrodeposition (**b**) and schematic representation of the NTNW electrode fabrication (**c**) (adapted with permission from ref. [216]. Copyright 2021 Elsevier).

Another example of using TeMs as templates was proposed by Amin et al., introducing a novel catalyst design utilizing nickel nanotube networks (Ni-NTNWs) integrated with nickel cobalt-layered double hydroxide (NiCo-LDH) nanosheets, aimed at enhancing electrochemical applications [216]. NiCo-LDH materials are known for their catalytic potential but suffer from low conductivity and agglomeration issues. The Ni-NTNWs provide a hierarchical nanostructured electrode architecture that combines high electrical conductivity, open macropores, and a dense array of active sites without the need for binders. The synthesis employs scalable techniques such as templating with track-etched membranes, electroless plating, and electrodeposition (Figure 26). The fabrication process of Ni-NTNWs involved several steps starting with the use of ion track-etched polycarbonate membranes with specific pore characteristics. The membranes were sensitized and activated with Pd nanoparticle seeds, involving immersion in solutions of SnCl_2_ and PdCl_2_/KCl sequentially to enhance nanoparticle density. Nickel electrodeposition (EP) followed using a bath containing NiSO_4_·7H_2_O and trisodium citrate dihydrate as the oxidation component, and DMAB as the reducing agent, forming a smooth and robust Ni film on the membrane. After deposition, the membranes were washed, and one side of the Ni-NTNWs was reinforced by additional Ni electrolyte application. To finalize, electrodes were isolated by attaching Cu wires, insulating the edges, and removing the polymer matrix with dichloromethane, resulting in exposed NTNWs ready for electrochemical experiments. Characterization confirmed the uniform coating of NiCo-LDH on both inner and outer surfaces of the nanotubes. The Ni-NTNW electrodes decorated with NiCo-LDH exhibited superior performance compared to pure Ni(OH)_2_ modifications for glucose sensing. The optimized catalyst layer, despite its thinness (20 µm), achieved high sensitivity (4.6 mA mM^−1^ cm^−2^), low detection limit (0.2 µM), rapid response time (5.3 s), excellent selectivity, and stability. The catalyst demonstrated linear detection ranges spanning four orders of magnitude, up to 2.5 mM analyte concentration. This study highlights the potential of interconnected metal nano-networks as effective, miniaturized catalyst electrodes and electrochemical sensors.

In a recent study by Shumskaya et al., ion track membranes were electrochemically modified by creating a metal-conductive polymer layer designed to monitor meat freshness through high sensitivity to ammonia, a key spoilage indicator. In this work, ion track membranes with 2 μm pores and a thin gold layer were used to electrochemically synthesize thin films from polyaniline (PANI) composites with gold (Au) or silver (Ag) nanoparticles. PET membranes with TM+PANI/Au and TM+PANI/Ag composites, featuring microstructured surfaces, were produced. Spectroscopic analysis confirmed that PANI was predominantly in its emeraldine salt form. These films demonstrated high sensitivity to ammonia, with detection limits of 40 mg/L for Au and 20 mg/L for Ag [217].

TeMs demonstrated remarkable versatility in the development of advanced sensor technologies. Their ability to create nanostructured materials with precisely controlled pore characteristics and functionalized surfaces enables highly sensitive and specific detection capabilities. TeMs were successfully integrated with various nanomaterials, such as metal nanotubes, nanowires, and layered structures, enhancing their electrochemical and optical properties. This led to significant advancements in biosensing, electrochemical sensing, and SERS, allowing for the detection of chemical and biological analytes at ultra-low concentrations. These innovations are particularly promising for clinical diagnostics, environmental monitoring, and other fields requiring precise analytical tools.

Looking ahead, several key areas offer promising avenues for further enhancing composite TeM-based sensor technologies. Innovations in material science could lead to the development of new composite structures with improved selectivity and sensitivity, along with enhanced mechanical, chemical, and thermal properties. Advanced surface modification techniques can expand the range of detectable analytes by enhancing selectivity and sensitivity. Integrating TeMs with microfluidic systems will facilitate rapid, on-chip analysis with minimal sample volumes, making sensors more portable and user-friendly. Additionally, the design of multiplexed sensors capable of simultaneous multi-analyte detection will improve diagnostic accuracy and efficiency. Efforts to scale up production processes will make these advanced sensors more cost-effective and accessible for widespread use in point-of-care diagnostics, environmental monitoring, and beyond. By addressing these areas, the potential of composite and hybrid CTeM-based sensors can be fully realized, opening the way for their extensive adoption across various scientific, industrial, and healthcare applications. The continued evolution of TeM- and CTeM-based technologies promises to drive significant advancements in analytical capabilities, ultimately leading to improved outcomes in diagnostics and monitoring, enhancing the quality of life globally.

### 4.3. Energy Storage Devices Based on CTeMs

Since the dawn of the 21st century, rapid technological advancements and a growing global population led to a significant increase in energy consumption, necessitating the development of efficient and capacious energy storage solutions. In this context, the advancement of energy storage systems emerged as a critical issue in the new global economy. Among various energy storage technologies, lithium-ion (Li-ion) batteries became the most widely used due to their high energy density and efficiency. However, current battery technologies, including Li-ion batteries, face several limitations. For instance, the energy density of Li-ion batteries, while high, still falls short of the demands for longer-lasting power in electric vehicles and portable electronics. Additionally, issues such as limited cycle life, thermal runaway risks, and safety concerns related to the flammability of liquid electrolytes hinder their widespread application. The formation of lithium dendrites during charge and discharge cycles can lead to short circuits and potential failures. Furthermore, the environmental impact of mining and disposing of lithium and other rare metals used in these batteries raises sustainability concerns. These challenges underscore the necessity for further developments in battery technology. Ongoing research in this field aims to enhance the performance of battery components and explore alternative devices. One promising avenue is the application of TeMs in batteries, which could revolutionize energy storage due to their tunable material properties, pore size, and ion fluency. Hybrid CTeMs, combining the benefits of various materials and structures, offer a potential solution to overcome the current limitations of conventional batteries. However, literature on the use of TeMs in energy storage devices remains sparse. This subsection reviews the latest studies on this topic, highlighting recent advancements and their implications for future research.

**Figure 27:**
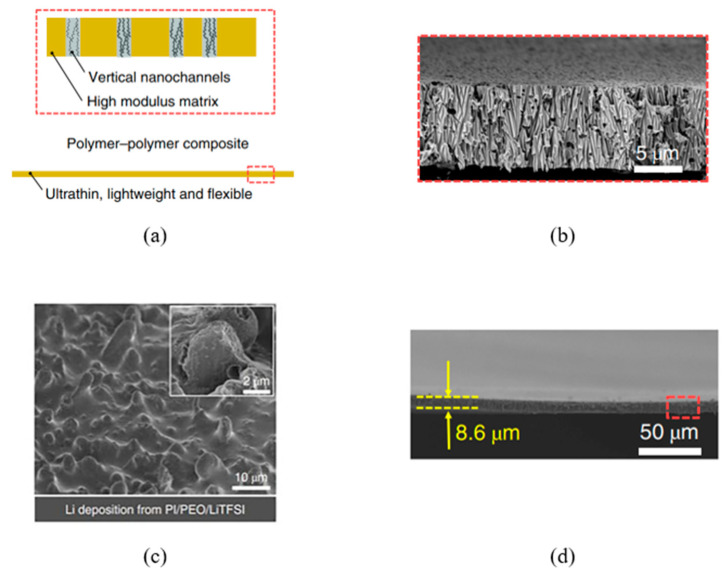
(**a**) Construction of PI/PEO/LiTFSI composite, (**b**) cross-sectional SEM image with zoomed-in aligned nanopores, (**c**) SEM image of PI/PEO/LiTFSI composite, (**d**) cross-sectional SEM image of the PI membrane (adapted with permission from ref. [219]. Copyright 2019 Springer Nature).

Li-ion batteries, which dominate the energy storage market, face challenges such as the formation of lithium dendrites during electrochemical plating and stripping processes [218]. These dendrites can penetrate the battery separator, leading to short circuits and potential failure. To address this issue, replacing liquid electrolytes with solid-state electrolytes was proposed. In 2019, Wan et al. introduced a novel approach using a polymer-polymer composite solid electrolyte [219]. They employed a commercially available Kapton film as a template to create aligned nanochannels through the track etching method (Figure 27a). The cross-sectional SEM image (Figure 27b) shows the prepared composite with detailed nanochannel structures, while Figure 27d reveals that the thickness of the polyimide (PI) membrane is approximately 8.6 µm. This composite solid electrolyte is nonflammable and contains lithium-ion conductive fillers, specifically PEO/lithium bis(trifluoromethanesulfonyl)imide (PEO/LiTFSI), which was deposited on the PI membrane through a simple dropping method using an acetonitrile solution (Figure 27c). According to the findings, compared with conventional solid polymer electrolytes based on PEO/Li salts, the hybrid electrolyte has five orders of magnitude higher modulus of elasticity and improved ionic conductivity. In addition, the pocket element assembled from the PI solid polymer electrolyte can still function when folded, twisted, and unfolded. Remarkably, even after undergoing nail and cutting tests, the pocket element successfully turned on an LED lamp, demonstrating its robustness and potential for flexible applications.

While various studies on batteries were proposed, it was not until 2021 that Muench et al. published a study aimed at using track membranes in fuel cells [220]. They claimed that creating a simple and safe separator for the fuel cell catalyst is important due to the current design problems related to mass activity and long-term stability [131]. According to their findings, much research suggested replacing and optimizing some components in the catalyst design [221,222,223]. However, Muench et al. developed a self-supported catalyst, which avoids the issues of carbon support corrosion and particle detachment commonly encountered in industrial fuel cell catalysts [224]. Thus, 1D ion track-etched polymer templates are viable materials for manufacturing parallelly aligned nanowires. TeMs can be a good alternative because increasing the length and decreasing the diameter of the nanowires leads to an increase in mass activity [225].

The goal of their research was to manufacture 3D nanowire networks with Ni@Pd nanoparticles embedded in the outer surface of the membrane. To achieve this, the PC membranes with a thickness of 25 µm and an average pore diameter of 400 nm were chosen as the template. Pd nanoparticles were deposited after swelling of the membrane in DMAB in a MeOH solution during activation in a PdCl_2_ solution. Ni nanoparticles were deposited through the electrochemical method using a Ni back electrode. The experimental results demonstrate that increasing the number of activation steps in the PdCl_2_ solution led to an increase in the amount of Pd. In terms of long-term stability properties, the authors compared the reference Pd@C catalyst with the Ni@Pd composite. The reference catalyst decomposed after 1000 cycles, whereas the Ni@Pd composite showed enhanced stability. The CV curves indicated that the synergistic effect between Pd and Ni allowed for the generation of extra OH ions for easier oxidation of MeOH at high concentrations [226]. Further ICP-OES analysis demonstrated that the dotted nanowire catalysts were not affected by the corrosion of the template.

**Figure 28:**
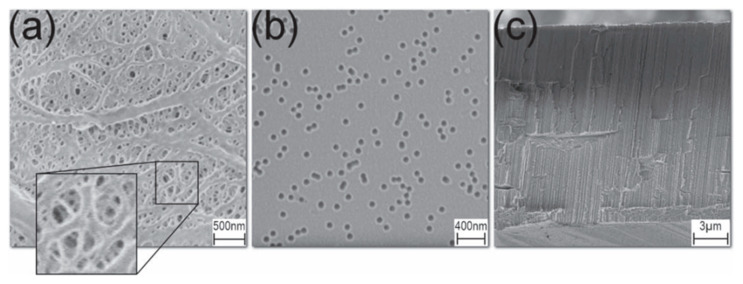
(**a**) SEM image of an SK Innovation separator, (**b**) surface of a PET TeM with an average pore diameter of approximately 100 nm and a pore density of 2.5 × 10^9^ cm^−2^, and (**c**) cross-section of the PET TeM (adapted with permission from ref. [227]. Copyright 2021 IOP Publishing Ltd. with license under CC 4.0).

The literature often focuses on the optimization of commercial batteries such as Li-ion batteries or fuel cells [226]. However, Lee et al. (2021) reported a study on the implementation of TeMs in Li-S batteries [227]. They claimed that Li-S batteries can be an alternative to Li-ion batteries due to their higher theoretical galvanic energy density of 2567 Wh kg^−1^ [228]. In practice, the Li-ion batteries face issues such as the polysulfide redox shuttle because of the soluble nature of the Li polysulfides [229]. Long-chain polysulfides are byproducts that can diffuse to the Li anode and react with it [230]. The resulting lower-order polysulfides, byproducts of the battery reactions, can diffuse to the Li anode and react with it, leading to self-discharge [231]. This issue can be mitigated through membrane or cathode modification. Lee et al. proposed using PET TeM with a thickness of 19 µm, ~100 nm pore diameters, and ion fluence between 10^7^ and 10^9^ ion cm^−2^ (Figure 28). To investigate the cycling performance of PET TeMs, the membranes were inserted between two SK Innovation separators in a Li-S coin cell. Electrochemical experiments demonstrated that after 15 cycles, the highest coulombic efficiency of 95% was reached with PET membranes having 115 nm pore diameter. Additionally, PET TeMs with low pore densities (1 × 10^7^ cm^−2^) limited the transport of lithium ions between the anode and cathode. The authors concluded that as the pore diameter decreases, the redox shuttle effect diminishes.

**Figure 29:**
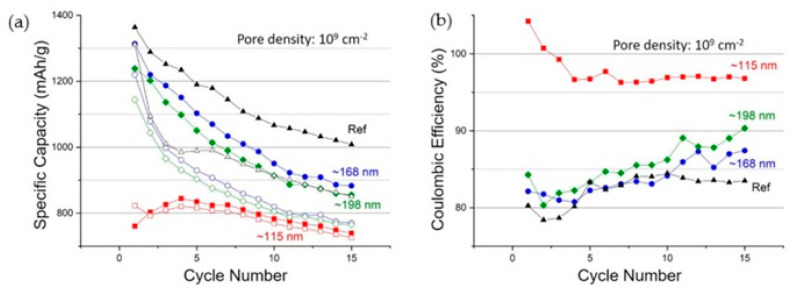
The cycling performance of lithium-sulfur coin cells utilizing PET etched ion track membranes, placed between two separators from SK Innovation, was assessed under a constant pore density (10^9^ cm^−2^) while varying the pore diameter. (**a**) Solid symbols represent charge capacities, and empty symbols indicate discharge capacities; (**b**) Coulombic efficiency as a function of pore size (adapted with permission from ref. [227]. Copyright 2021 IOP Publishing Ltd. with license under CC 4.0).

To assess the cycling performance of PET TeMs, the membranes were placed between two SK Innovation separators in a Li-S coin cell. Electrochemical tests revealed that, after 15 cycles, PET membranes with a 115 nm pore diameter achieved the highest coulombic efficiency of 95% (Figure 29). Additionally, PET TeMs with low pore densities (1 × 10^7^ cm^−2^) restricted lithium ion transport between the anode and cathode. The authors concluded that as pore diameter decreases, the redox shuttle effect is reduced. High porosity leads to better lithium conductivity and coulombic efficiency. The 115 nm TeMs exhibited the highest coulombic efficiency for several reasons already discussed, one being the ion selectivity of PET TeMs.

Electrochemically deposited iron nanotubes were studied as anode material for lithium-ion batteries [232]. The study showed that the complete degradation of the nanotubes occurred after 492 cycles when operating under a charge capacity limit of 1000 mA h/g, commonly used in lithium-ion batteries. Decreases in discharge capacity began around the 380th cycle, coinciding with the onset of nanostructure degradation due to the formation of amorphous inclusions and increased macrostress and structural distortion. Continued cycling resulted in more amorphous inclusions in the nanotube structure. By the 492nd cycle, characterized by the most significant drop in discharge capacity, the nanotube surface was covered with feather-like growths, indicating the complete degradation of the structure.

**Figure 30:**
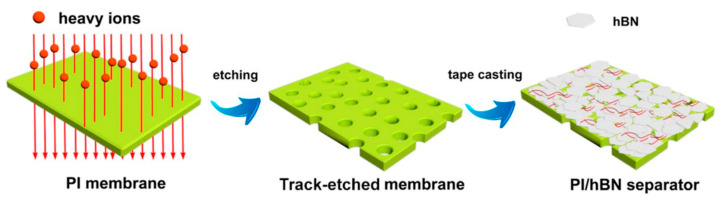
Schematic representation of the PI/hBN separator fabrication process (adapted with permission from ref. [236]. Copyright 2022 American Chemical Society).

Another problem with Li-ion batteries concerns the thermal stability and conductivity of separators [233]. Currently, separators based on the PE and PP membranes are common in Li-ion batteries, with melting points of ~135 °C and ~165 °C, respectively [234]. Prolonged use can cause the micropores in PP or PE separators can collapse near their melting points, making the membrane insulating rather than ionically conductive [235]. A potential solution is to coat the polymer matrix with organic molecules or synthesize ceramic composite membranes [234]. Liu et al. proposed using TeMs as separators to improve the thermal stability of the battery [236]. PI foils with 14 µm thickness were irradiated by heavy ions (2 × 10^8^ ion cm^−2^) to create nanochannels (Figure 30). After irradiation, the PI samples were etched in NaOCl solution to form 600 nm pore diameters. Finally, a 3 µm layer of hexagonal boron nitride (hBN) layer was deposited using the doctor blade method. Hersam et al. reported that composite separators with hBN nanosheets and PVDF on carbon templates improved the electrochemical characteristics, prompting the choice of hBN as a coating material for the PI TeM [237].

**Figure 31:**
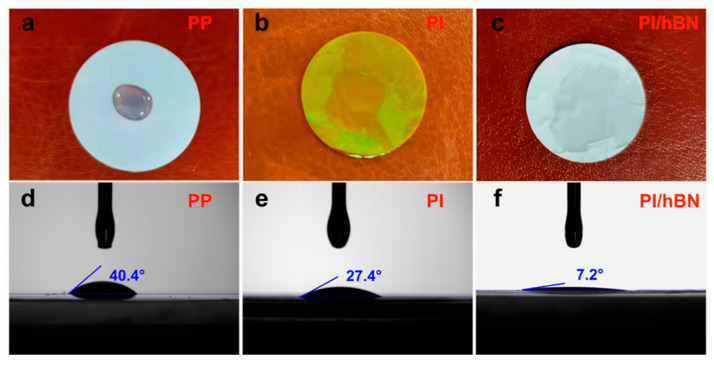
Images of various separators after the addition of electrolyte on the surfaces: (**a**) PP, (**b**) PI TeM, and (**c**) PI/hBN. Contact angle experiments between separators and electrolytes: (**d**) PP, (**e**) PI TeM, and (**f**) PI/hBN separator (adapted with permission from ref. [236]. Copyright 2022 American Chemical Society).

The investigation of physical properties demonstrated the high quality of the prepared PI/hBN separator. Key criteria for good separators include porosity, permeability, and electrolyte uptake, all of which enhance the transport of Li ions and improve ionic conductivity. The uncoated PI TeM had a porosity value of 32.6%, while PI/hBN was higher at 44.5%. The air permeability of the PI/hBN by Gurley value is equal to 33.4, compared to 11.5 for the PI TeM, indicating better electric resistance for the prepared separator. In terms of electrolyte uptake, the PI/hBN separator achieved 62.7%, attributed to the affinity of hBN for the electrolyte and good permeability. The comparative results on the wettability of the PI/hBN separator and PP showed that the PI/hBN separator had the lowest values of contact angle and wettability, due to its high affinity for the electrolyte solution and a high number of pores (Figure 31). The tensile strength of the prepared separator demonstrated isotropic behavior, with a tensile straight value of 220.1 MPa and Young’s modulus of 1460.2 Pa, higher than that of the PP-based separator at 440.1 MPa. These values help prevent the PI/hBN separator from degradation and short circuits in the battery.

In conclusion, the integration of TeMs, including CTeMs, in energy storage devices presents a promising frontier in battery technology. Studies using various TeMs-based materials such as PET, PI, and PC as templates for solid electrolytes, Li-S batteries, and separators for fuel cells and Li-ion batteries show significant improvements in performance and stability. TeMs offer advantages such as higher ionic conductivity, improved thermal stability, and increased mechanical strength. Their robustness, flexibility, and varied porosity can enhance conductivity and prevent short circuits.

The incorporation of nanomaterials such as graphene and metallic nanoparticles into CTeMs may further enhance their potential in energy applications. Graphene, for instance, can improve electrical conductivity and mechanical properties, while metallic nanoparticles can enhance catalytic activity and surface area, thereby improving overall battery performance. Despite these prospects, research in this area is still in its early stages. Future studies should focus on optimizing fabrication processes to enhance uniformity and scalability of CTeMs. Additionally, exploring the compatibility of CTeMs with different electrolyte compositions and electrode materials could further broaden their applicability in diverse energy storage systems. Overall, the integration of TeMs and CTeMs into energy storage devices holds great promise for developing more efficient, durable, and safe batteries. These innovations have the potential to revolutionize the energy storage industry, opening the way for advancements that meet the growing demand for sustainable energy solutions.

### 4.4. Biomedical Applications of Composite Track-Etched Membranes

CTeMs may demonstrate versatile potential beyond environmental, sensing, and energy storage applications, particularly in biomedical and separation applications. In biomedicine, these membranes may offer precise molecular filtration and catalytic capabilities for synthesis, purification, or detection, leveraging their customizable surface functionalities and high selectivity. In separation technologies, CTeMs may hold promise particularly for efficient separation of oil–water mixtures and biomolecules, benefiting pharmaceutical purification and water treatment. Their adaptability and robust performance may underscore their potential to drive innovations in biomedical diagnostics and industrial separation processes. This section of the review will explore additional promising application areas of CTeMs.

Polymeric membranes became pivotal in separation processes across diverse industrial and environmental applications, owing to their adaptability, ease of fabrication, and customizable properties. Their intrinsic flexibility, chemical resistance, and amenability to modification with functional groups or nanoparticles enable them to achieve high selectivity and permeability in separating gases, liquids, and solutes. Techniques such as track etching and electrospinning further enhance membrane performance by creating precise pore structures and incorporating functional materials, crucial for applications such as membrane distillation (MD), gas separation, and micro/ultrafiltration.

In gas separation technology, TeMs play a critical role by leveraging mechanisms such as affinity and size selectivity. Affinity selectivity involves surface modification or the integration of nanoparticles that selectively interact with specific gas molecules. Early studies, such as those by Acharya et al. in 2006, explored track-etched polymeric membranes enhanced with titanium (Ti) deposition to improve hydrogen permeability. By vacuum-evaporating a thin Ti film onto polymer membranes and subsequently characterizing them via UV-vis spectroscopy and optical microscopy, enhanced hydrogen permeation and selectivity were confirmed. Ion beam irradiation and chemical etching techniques were also employed to develop high-permeation track-etched membranes, with selective Ti deposition significantly enhancing hydrogen permeation while restricting other gases. These findings underscore polycarbonate (PC) as an effective material for such membranes [154].

Palladium (Pd) nanoparticles are highly effective in enhancing hydrogen separation when integrated into track-etched membranes. This integration significantly improves both hydrogen permeability and selectivity over CO_2_ and N_2_ gases. Kamakshi et al. investigated the functionalization of PET TeM with carboxylic and amino groups. They synthesized 5 nm diameter Pd nanoparticles and deposited them onto the membranes’ pore walls and surfaces, observing that aminated membranes exhibited stronger binding of Pd nanoparticles compared to non-functionalized membranes [152]. The study highlighted the role of surface functionalization in modifying the membrane’s gas permeability and selectivity, particularly enhancing hydrogen separation technology. This affinity was previously discussed by Awasthi et al. in 2014 [238]. Further research by Kamakshi et al. explored how the deposition time of Pd nanoparticles influences gas separation efficiency. They found that increasing deposition time enhances the selectivity of hydrogen over CO_2_ and N_2_, underscoring the importance of nanoparticle density in optimizing membrane performance for high-purity hydrogen applications [239].

In a separate study, Kumar et al. (2021) developed hydrogen-selective membranes by incorporating Pd nanoparticles into PC TeMs using UV irradiation. This method effectively enhanced membrane selectivity and permeability for hydrogen gas while maintaining high discrimination against CO_2_ and N_2_. The study emphasized the significance of uniform nanoparticle distribution achieved through controlled UV exposure followed by immersion in a Pd nanoparticle solution, thereby enhancing the membrane’s active sites and improving hydrogen selectivity [240].

**Figure 32:**
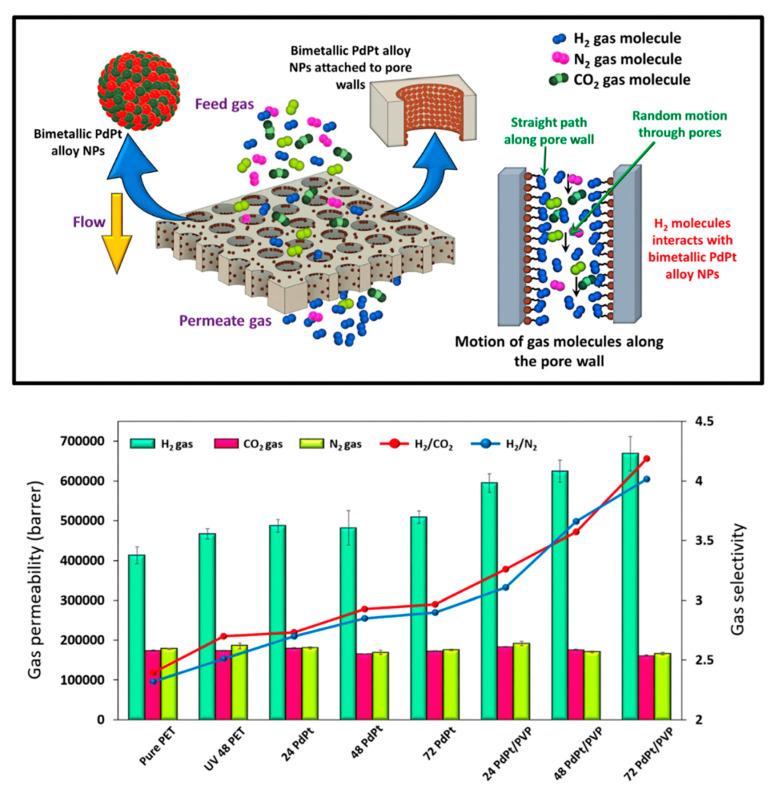
Gas separation mechanism of PdPt BNP decorated track-etched polymer membranes and gas separation performance of the UV functionalized PdPt BNP dipped PET membrane series (adapted with permission from ref. [241]. Copyright 2024 Royal Society of Chemistry with license under CC BY-NC 3.0).

Recently, Saini et al. proposed enhancing hydrogen separation properties in PET TeMs by synthesizing palladium-platinum (Pd-Pt) bimetallic nanoparticles (BNPs) [241]. They deposited these BNPs, approximately 8 nm in size, onto the membranes over varying time intervals up to 72 h. Techniques such as UV functionalization and the use of polyvinylpyrrolidone (PVP) as a binder were employed to enhance nanoparticle adhesion. The study demonstrated significant improvements in gas separation properties, including a marked increase in hydrogen permeability and selectivity over CO_2_ and N_2_ gases (Figure 32).

The maximum permeability for H_2_ gas reached a 668,128 barrier, marking a 61% increase. Additionally, the selectivity between H_2_ and CO_2_ rose from 2.39 to 4.18, a significant increase of 74.89%, while the selectivity between H_2_ and N_2_ increased from 2.31 to 4.01, showing a substantial improvement of 73.59%. These results underscore the effectiveness of PdPt BNP decoration in enhancing both permeability and selectivity for hydrogen gas separation applications [241]. These studies collectively highlight the effectiveness of Pd nanoparticles and Pd-Pt bimetallic nanoparticles in enhancing the performance of TeMs for hydrogen separation applications, leveraging surface functionalization and controlled deposition techniques to achieve superior gas permeability and selectivity.

While TeMs were extensively studied and applied in various separation processes, such as oil–water separation [7,8,57] and the isolation of water-extractable soil colloids [242], as well as in gas and ion transport [243], the application of CTeMs for these purposes remains relatively unexplored. Further research into leveraging CTeMs could uncover novel approaches to improve selectivity, efficiency, permeability, and durability in these separation applications, potentially opening new avenues for environmental remediation and industrial processes.

In the biomedical realm, the appeal of nanomaterials grew significantly over the last decade due to their unique characteristics such as small size, high surface area, durability, flexibility, and chemical reactivity. These characteristics enable nanomaterials to interact with biological systems at the molecular level, making them ideal candidates for biomedical applications. Their small size allows them to penetrate biological barriers and reach targeted tissues and cells more effectively than larger particles. This ability is particularly beneficial for drug delivery systems, as nanomaterials can be engineered to deliver therapeutic agents directly to diseased cells, reducing side effects and improving treatment efficacy. Their high surface area provides ample space for functionalization with various biomolecules, enhancing their specificity and interaction with biological targets [244]. Durability and flexibility are also critical features, as biomedical devices and materials need to withstand the dynamic and often harsh environments within the body. Nanomaterials’ chemical reactivity allows for the creation of complex structures and surfaces that can interact with biological molecules in specific ways, enabling the development of advanced diagnostic and therapeutic tools [245].

Despite these advantages, the exploration of nanotechnologies in biomedical contexts, particularly concerning the integration of nanoparticles and thin films, remains relatively limited. TeMs emerged as crucial components in this pursuit, serving as versatile platforms for the development of biomedical devices and materials. TeMs act as the polymer matrix for the deposition of metals on one side, including noble metals, to create (bi)metallic nanomaterials, and also serve as a modifiable platform that can be functionalized with various chemicals, including ligands and biomarkers. These nanomaterials combine their structural benefits with the unique properties imparted by each functional component. Nanochannels offer unparalleled functionality in the sensing realm due to their ability to precisely modulate ionic currents in response to various stimuli. By tailoring the surface properties of nanochannels, they can be engineered to detect a wide range of compounds, from small ions to complex biomolecules such as DNA and proteins. For instance, single nanochannels demonstrated exceptional sensitivity and specificity in detecting low-abundance biomarkers, such as microRNAs (miRNAs) associated with diseases such as liver cancer, with detection limits reaching as low as 97.2 aM [246]. Furthermore, the unique ability of nanochannels to switch between “on” and “off” states in response to molecular interactions makes them ideal for real-time monitoring of cellular processes, offering valuable insights for diagnostics and therapeutic interventions [247]. These advanced sensing capabilities underscore the potential of nanochannels to revolutionize biomedical diagnostics and open new horizons in the development of smart, responsive biosensors.

**Figure 33:**
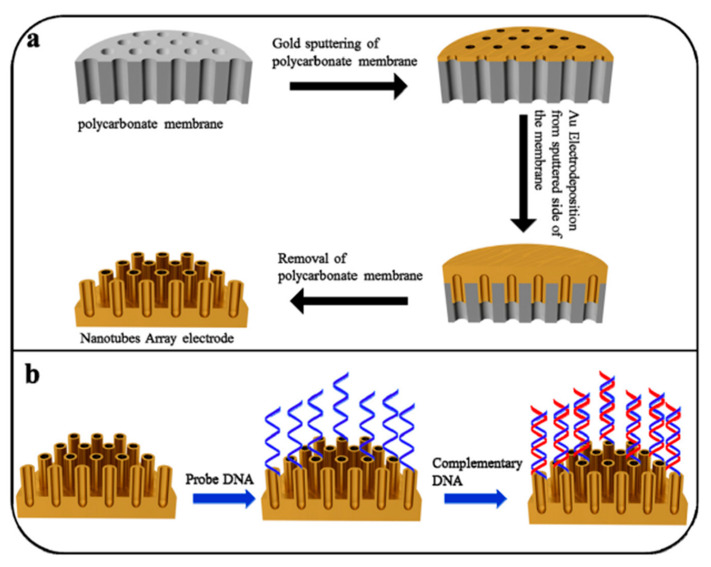
(**a**) schematic of AuNTs synthesis and (**b**) DNA biosensor setup using AuNTs electrodes. Biosensor includes working electrode (WE), reference electrode (RE), and counter electrode (CE). AuNTs array electrodes showed improved electron transfer compared to bare Au electrodes. Biosensor detected DNA in linear range of 0.01 ng/µL to 100 ng/µL, with a limit of detection of 0.05 ng/µL (adapted with permission from ref. [33]. Copyright 2016 Elsevier).

In 2016, Torati et al. suggested an electrochemical biosensor for detecting *Mycobacterium tuberculosis* DNA [33]. The AuNTs array was synthesized via the electrochemical deposition method on the PC membrane (200 nm diameter) (Figure 33a). The DNA biosensor was constructed using AuNTs electrodes, as depicted in Figure 31b. To immobilize the probe DNA on the AuNTs electrode, a 10 μL droplet of 100 ng/mL probe DNA solution in Tris-EDTA buffer was applied to the electrode surface for 12 h. To initiate the hybridization process, 10 μL of complementary DNA with different concentrations was carefully dropped onto the electrode surface immobilized with the probe. The bioelectrodes were then subjected to an incubation period of 45 min at 37 °C. After hybridization, the bioelectrodes were rinsed with Tris-EDTA buffer and stored at 4 °C when not actively utilized. Compared to bare Au electrodes, Au NTs array electrodes demonstrated better electron transfer. The synthesized DNA biosensor showed a linear range from 0.01 ng/µL to 100 ng/µL with a limit of detection of 0.05 ng/µL.

CTeMs may potentially revolutionize separation technologies by combining the inherent benefits of track-etched membranes with advanced functionalities enabled by nanostructured materials. Ongoing research suggests promising advancements in their application across diverse separation technologies. Adjusting composition uniformity, controlling pore size, and enhancing surface functionality could significantly broaden their scope, potentially enabling tailored CTeMs capable of precisely filtering specific biomolecules such as proteins or nucleic acids, thus offering new possibilities in biomedical research. Additionally, custom-configured CTeMs may enhance the efficiency of separating challenging molecules such as enantiomers, utilizing materials such as metal-organic frameworks (MOFs), aptamers, or antibodies for improved specificity.

In industrial applications, optimizing production processes to improve the cost-effectiveness and scalability of CTeMs could enhance their utility in separating complex gas mixtures and treating industrial wastes. Notably, CTeM nanostructures may exhibit catalytic properties, facilitating both separation and chemical transformations, which could be advantageous for environmental and industrial applications. As demand for advanced separation technologies grows, CTeMs are positioned to play pivotal roles across scientific, industrial, and environmental sectors, offering innovative solutions to complex separation challenges.

TeMs already demonstrated effectiveness in biomedical applications, where they were successfully modified with biomolecules through organic reactions. Single and multiple nanochannels are emerging as promising tools for selective ion transport, DNA sequencing, and biosensing due to their high sensitivity and specificity in detecting low-abundance biomarkers. TeMs also showed versatility in biomedical research, including applications such as bone regeneration through nanotube exploration.

However, the integration of CTeMs in the biomedical field is still in its early stages and lacks extensive proven applications. Current research highlights their potential, but significant challenges remain to be addressed. Future efforts should focus on optimizing CTeM functionalization, improving biocompatibility, and scaling up production for commercial use. Continued exploration and development of CTeMs hold immense promise for advancing healthcare technologies, potentially opening new frontiers in biomedical science and engineering.

## 5. Conclusions, Future Directions, and Prospects for Composite Track-Etched Membranes (CTeMs)

CTeMs represent a significant advancement in membrane technology, offering enhanced performance and versatility across a variety of applications. These specialized membranes combine the properties of traditional track-etched membranes with additional functional phases, such as metal or metal oxide nanoparticles, resulting in a multifunctional structure tailored for specific uses. The development of CTeMs marks a transformative step in addressing the limitations of traditional membrane technologies, particularly in areas requiring high selectivity, sensitivity, and efficiency.

In energy storage, CTeMs demonstrate a remarkable ability to address several critical issues plaguing current technologies. For instance, in lithium-ion (Li-ion) batteries, the formation of lithium dendrites during charge and discharge cycles can lead to short circuits and potential failures. Traditional batteries also face significant limitations regarding shape, flexibility, thermal stability, energy density, and chemical safety. By modifying the porous nature of durable track-etched membrane materials such as polyimide or Kapton with conductive and catalytic nanostructures, CTeMs can improve ion transport and charge–discharge cycles in batteries and supercapacitors while providing flexibility and mechanical durability. This advancement may enhance the energy density and lifespan of energy storage devices and facilitate the development of more sustainable and efficient energy storage solutions. The nonflammable nature of composite solid electrolytes further enhances safety, making future generations of CTeMs ideal for applications in electric vehicles and portable electronics. The robustness and flexibility of these membranes were already illustrated in studies where composite solid electrolytes maintained functionality even after undergoing physical deformation, such as folding and twisting. This resilience is particularly critical for developing wearable energy storage devices, which are becoming increasingly important in modern technology.

In sensor technology, CTeMs offer a promising solution to the persistent challenges of limited sensitivity and selectivity, slow response times, and stability issues under varying conditions. These membranes, which combine multiple functionalities within a single structure, can achieve high selectivity and sensitivity, crucial for accurately monitoring specific molecules or ions in environmental monitoring and medical diagnostics. By tailoring the surface of CTeMs with functional groups or incorporating nanoparticles and conductive layers, their specificity and sensitivity can be significantly enhanced, enabling the detection of low analyte concentrations. The integration of nanomaterials such as metallic nanoparticles and carbon-based nanomaterials further extends the functionalities of CTeMs, improving electrical conductivity, catalytic activity, and surface area for better signal transduction. This capability is further amplified by the robust polymer matrices of CTeMs, ensuring durable and stable performance in diverse environments. Consequently, CTeMs are poised to revolutionize the development of next-generation sensors, addressing critical needs in healthcare, environmental monitoring, and industrial applications.

The environmental applications of CTeMs are equally promising. Traditional sensors and membranes often struggle with the detection and removal of pollutants with high precision. In contrast, CTeMs, with their customizable properties, offer a robust solution for environmental monitoring and remediation. For instance, incorporating reactive nanoparticles on the membrane walls can significantly enhance mass transfer and absorption capacity. These nanoparticles can also provide catalytic activity, decomposing pollutants into non-toxic or less toxic species, thus achieving both separation and elimination simultaneously. This strategy improves the efficiency of capturing and eliminating pollutants such as heavy metals, organic pollutants, and other hazardous substances from water and air, contributing to a cleaner and safer environment. Their promise is in catalytic applications, where high surface area and catalytic activity are paramount. The incorporation of catalytic nanoparticles within the membrane matrix enhances their efficiency, particularly in repeated use, by providing a large and stable substrate for the stabilization of these active phases. This makes CTeMs ideal for various chemical reactions and industrial processes. The immobilization of enzymes onto porous membranes can provide membrane reactors with improved productivity and reduced enzyme deactivation issues, highlighting their potential in biotechnology and chemical engineering applications. The customization of CTeMs for specific catalytic processes underscores their versatility and effectiveness in enhancing reaction rates and product yields.

**Figure 34:**
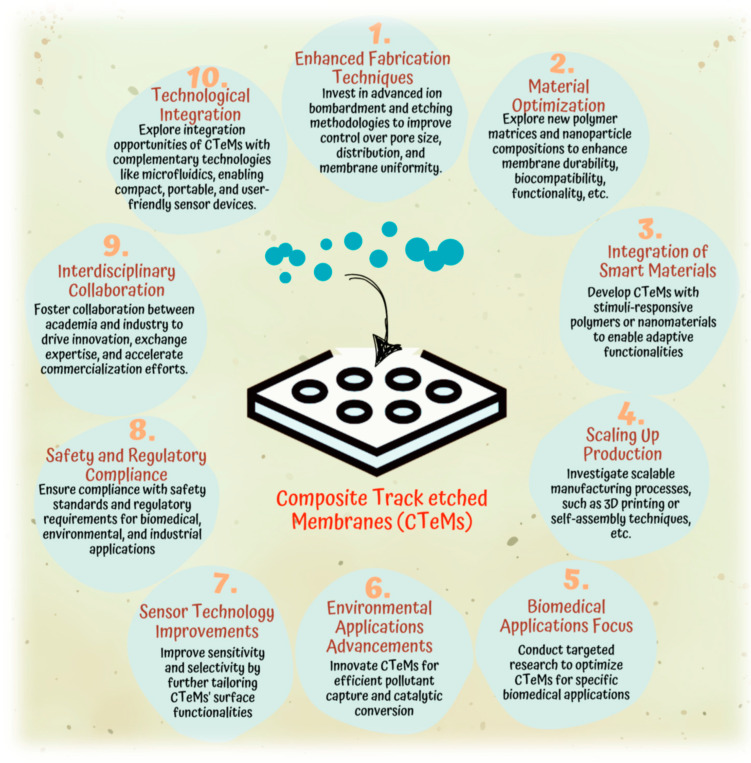
Measures for the further development and adaptation of CTeMs. The figure summarizes critical steps to advance CTeM technology, including enhanced fabrication techniques, material optimization, integration of smart materials, scaling up production, focus on promising applications, sensor technology improvements, ensuring safety and regulatory compliance, fostering interdisciplinary collaboration, and exploring technological integration.

In biomedical applications, CTeMs may offer unique advantages for drug delivery, tissue engineering, and diagnostic devices. Their ability to be functionalized with biocompatible materials and tailored for specific biological interactions makes them ideal for targeted drug delivery systems, where precise control over drug release is crucial, such as in wound dressings. Additionally, the high surface area and porosity of CTeMs can facilitate the growth and proliferation of cells, making them suitable for tissue engineering scaffolds. The integration of stimuli-responsive materials into CTeMs further expands their potential, enabling the development of smart membranes that can respond to environmental triggers such as pH, temperature, and light, enhancing their functionality in biomedical applications. Moreover, in combination with energy storage devices, future wearable electronics for health monitoring could utilize these composite porous membranes. Figure 34 illustrates key measures for advancing the development and adaptation of CTeMs across various applications. These measures encompass technological innovations and strategic approaches aimed at enhancing membrane performance, functionality, and integration into various fields such as biomedical, energy, and sensor technologies.

Despite their numerous advantages, the development and application of CTeMs are not without challenges. The fabrication process relies on sophisticated ion bombardment methodologies, which can be cost-intensive and technically demanding. Precise control over pore size and distribution requires advanced techniques and meticulous optimization, presenting significant technological difficulties. Additionally, the biocompatibility and limitations in polymer matrix choices pose challenges. Traditional polymer matrix choices such as PET or PC must give way to biodegradable and biocompatible polymers to facilitate their integration into various fields. This shift necessitates overcoming technical hurdles and achieving technological improvements, particularly in ensuring that these new materials meet the rigorous standards required for production methodologies. Ensuring uniform distribution and strong adhesion of composite materials within the membrane matrix is critical to maintain performance consistency and durability of CTeMs. The potential of these membranes is vast, but their widespread adoption in various industries hinges on overcoming these challenges. Additionally, the scalability of production and the cost-effectiveness of the materials used are crucial factors that must be addressed for CTeMs to become commercially viable.

A critical consideration in the wider adoption of CTeMs is their cost-effectiveness. Although the initial production costs are high due to complex methodologies, CTeMs may offer long-term savings through their durability and efficiency, particularly in filtration and separation applications. Their extended lifespan and lower maintenance needs can offset the upfront investment. Additionally, as production technologies improve and the demand for CTeMs increases, economies of scale are expected to reduce unit costs, making them more affordable for broader industrial use. Enhanced process efficiency and minimized waste also contribute to their economic appeal.

Looking to the future, the potential of CTeMs is expansive, with ongoing research and development aimed at overcoming current limitations and expanding their applications. Advances in nanotechnology, materials science, microfluidic systems, and fabrication techniques will likely lead to even more sophisticated and high-performance CTeMs. Innovations such as 3D printing and self-assembly techniques could provide new pathways for fabricating CTeMs with intricate structures and functionalities, transforming these porous composite membranes into sophisticated devices with rapid, on-chip, portable, and user-friendly capabilities. The exploration of new materials and functionalization methods will further enhance the capabilities of CTeMs, making them more adaptable and efficient for a broader range of applications. For instance, incorporating biodegradable polymers can make CTeMs more suitable for biomedical applications. Collaboration between academia, industry, and government agencies will be crucial in driving innovation and facilitating the commercialization of CTeMs, ensuring that their benefits can be realized across different sectors. This concerted effort will help in overcoming existing challenges and unlocking the full potential of CTeMs, facilitating their integration into everyday technologies and advanced industrial processes.

In conclusion, composite track-etched membranes represent a significant advancement in membrane technology, offering enhanced performance and versatility across various applications. Their ability to address the limitations of traditional membranes and provide solutions for challenges in sensor technology, environmental monitoring, energy storage, catalytic processes, biomedical applications, and more underscores their transformative potential. As research and development continue to advance, CTeMs are poised to play a critical role in driving innovation and addressing the evolving needs of modern society.

## Figures and Tables

**Table 1:** Functionalization of PET TeMs by grafting.

Grafted Monomer	Added functionality	Refs.
2-dimethylaminoethyl methacrylate	pH and temperature responsivity	[43]
Glycidyl methacrylate	Epoxy functionality	[44]
*N*-isopropylacrylamide	Temperature responsivity	[45]
Styrene	Hydrophobization for purification of saline solutions	[46]
Triethoxyvinylsilane and 1H,1H,2H,2H-perfluorododecyltrichlorosilane	Pesticide (carbendazim) removal	[47]
Triethoxyvinylsilane	Water desalination	[48]
Triethoxyvinylsilane and styrene	Membrane distillation of radioactive wastes.	[49]
Acrylic acid	Detection of sub-ppb concentrations of Pb^2+^	[37]
Stearyl methacrylate	Oil–water separation	[50]
*N*-vinylcaprolactam	Temperature responsivity	[51]
Glycidyl methacrylate and acrylic acid	Selective functionalization	[52]
Glycidyl methacrylate	Fluorescence-based ph biosensing	[53]
*N*-isopropylacrylamide	Temperature responsivity	[54]
Acrylic acid and 4-vinylpyridine	Detection of heavy metal ions	[55]
Acrylic acid	pH responsivity, reactive layer for further functionalization	[56]
Propyltrichlorosilane	Oil/water separation	[57]
Acrylic acid and di(ethylene glycol)methyl ether methacrylate	Separation of alkaline ions	[58]
2-hydroxyethyl-methacrylate and N-isopropylacrylamide	Environmental responsiveness	[59]

**Table 2:** Typical etching conditions for the different types of TeMs.

Polymer	Etching Solution		Sensibilization	Deactivator	T, °C	Etching Time, d = 1 µm, h	Etching Selectivity
PC	NaOH		UV	Methanol	50	1	100–10,000
PET	NaOH		UV, DMF	Methanol	50	1	10–1000
	Na_2_CO_3_				80	1	1000
PP	CrO_3_	H_2_SO_4_			80	1	10–100
PVDF	KMnO_4_	NaOH			80	5	10–100
PI	NaOCl	Na_2_B_4_O_7_			50	1	10–1000

PC—polycarbonate, PET—polyethylene terephthalate, PP—polypropelene, PVDF—polyvinylidene fluoride, and PI—polyimide.

**Table 3:** CTeMs featuring various nanostructures, including nanowires (NWs), nanotubes (NTs), and nanoparticles (NPs), and synthesized using different deposition techniques.

Deposited Materials	Structure	Polymer Template			Synthesis Approach	Application of CTeM	Refs.
		Pores Size and Density	Substrate	Thickness, µm			
Ni and Pd	Ni NWs and Pd NPs	400 nm; 1.5 × 10^8^ cm^−2^	PC	25.0	Electroplating	Catalyst for fuel cell	[131]
ZnO	NWs	90 nm; 1.0 × 10^9^	PC	21.0	Electroplating	-	[132]
Au	NWs	80 and 170 nm; 4.0 × 10^8^ cm^−2^	PC	30.0	Electroplating	Catalysis	[133]
Cu	NWs	136 ± 6 nm; 8.0 × 10^8^ cm^−2^	PC	30.0	Electroplating	CO_2_ reduction	[134]
	NTs	380 ± 20 nm; 4 × 10^7^ cm^−2^	PET	12.0		-	[135]
Pt	Nanocones	550 nm; 3.0 × 10^7^ and 1.0 × 10^8^ cm^−2^	PI and PC	12.0 50.0	Electroplating	Electrocatalysis	[136]
Ni@Au	NTs	380 ± 20 nm; 4 × 10^7^ cm^−2^	PET	12.0	Electroless/Electroplating	Catalysis Antioxidants Antimicrobial activity SERS	[102,103,130]
Ag	NWs	60–200 nm; 8.4 × 10^9^, 1.2 × 10^9^, 4 × 10^8^ cm^−2^.	PET	-	Electroplating	Sensors	[137]
Co/CoCo_2_O_4_	NWs	400 nm; 4 × 10^7^ cm^−2^	PET	12.0	Electroplating	CO adsorption, Catalysis	[108]
FeCo–Fe_2_CoO_4_/ Co_3_O_4_	NWs	400 nm; 4 × 10^7^ cm^−2^	PET	12.0	Electroplating	Catalysis	[138]
Pd@Pt	NTs	400 nm; 1.5 × 10^8^ cm^−2^,	PC	20.0	Electroless	Electro-oxidation of methanol	[115]
Ir	NPs	400 nm; 1.5 × 10^8^ cm^−2^	PC	25.0	Electroless	Catalysis	[139]
Cu	NTs	390–400 nm; 4 × 10^7^ cm^−2^	PET	12.0	Electroless	Catalysis Heavy metal ion sorption	[119,122,124,140]
Pd	NPs	410 nm; 4 × 10^7^ cm^−2^	PVP-*g*-PET	12.0	Electroless	Catalysis	[66]
NiFe@Au	Nanobeads	80 nm; 5 × 10^7^ cm^−2^	PET	11.0	Electroless	Immunocapture of nanocarriers	[130]
Protein@Au	NTs	400 nm;	PC	10.0	Electroless	Biodevices in biochemical and biomedical application	[141]
Au Ag	NTs	400 nm	PET	12.0	Electroless	Catalysis	[117,142,143]
Sr(CaP)	NTs	200 nm;	PC	20.0	Electroless	Biomedical	[144]
NiCo	NTs	650 nm; 1.0 × 10^8^ cm^−2^	PC	30.0	Electroless	Catalysis	[145]
Ni_2_O_3_@Cu	NTs	400 nm 4.0 × 10^7^ cm^−2^	PC	20.0	Electroless	Catalysis	[125]
Au	Nanoflowers	50 nm	PC	20.0	Electroless	SERS sensor	[146]
Au	NTs	30 nm; 6.0 × 10^8^ cm^−2^	PC	10.0	Electroless	Molecular sieving	[147]
Rh	NTs	100–400 nm; 1 × 10^8^ cm^−2^	PC	30.0	Electroless	Catalysis	[148]
Bi	NTs	2.5 × 10^8^ cm^−2^	PC	25.0	Electroless	CO_2_ reduction, heavy metal sensing	[149]
Ir@Bi	NTs	400 nm	PC	25.0	Electroless	Degradation of dyes; Pb (II) sensing	[150]
Pt	NPs	500 nm; 3 × 10^7^ cm^−2^	PI	2.0	Radiation-Induced	-	[151]
Pd	NPs	100–200 nm;	PET	-	Electroless	Gas separation	[152]
Cu/CuO	NTs	430 nm; 4 × 10^7^ cm^−2^	PET	12.0	Electroless	Catalysis Heavy metal ion sorption	[32]
Au, Ag	NPs	400 nm	PAA-g-PET	12.0	Radiation-induced	Catalysis	[64]
Pt	NPs	500 nm; 3 × 10^7^ cm^−2^	PI	2.0	Radiation-induced	-	[151]
Cu(OH)_2_ Cu	NPs	400 nm	PAA-g-PET	12.0	Radiation-induced	Catalysis	[128]
HKUST-1	NPs	300 nm; 5 × 10^8^ cm^−2^	PET	12.0	Layer-by-layer coating	Separation	[153]
Ti	NPs	100–150 nm	PC, PET	30.0 25.0	Vacuum evaporation (10^−6^ Torr)	H_2_ purification	[154]

**Table 4:** The effect of deposition time on the rate constant of decomposition of *p*-NP by Au/Ag/PET and Au/PET catalysts [155].

Deposition Time, h	Rate Constant, min^−1^	
	AS_I_ (Au/Ag/PET)	AS_II_ (Au/PET)
1	0.087 ± 0.02	0.041 ± 0.01
5	0.084 ± 0.01	0.074 ± 0.02
24	0.082 ± 0.005	0.05 ± 0.01

**Table 5:** Reusability and performance of Ag/PET composite membranes in the reduction in *p*-nitrophenol at 40 °C [29].

Cycles	Apparent Constant Rate [k × 10^−3^ min^−1^] *p*-NP to *p*-AP	Conversion of Initial Reagent *p*-NP to *p*-AP
1st	85.0	89.8%
2nd	69.3	87.1%
3rd	66.5	80.3%

**Table 6:** Reaction rates (*k*), conversions and activation energies (E_a_) for decomposition of *p*-NP by CuNTs@PET TeM catalyst in flow and static modes [157].

Test Mode	Test Run	k × 10^−2^, min^−1^	*p*-NP Conversion	E_a_, kJ/mol
Static	1	5.1 ± 0.4	98.4 ± 4.1	28.32
	2	3.6 ± 0.2	95.0 ± 3.2	
	3	3.5 ± 0.3	88.2 ± 4.0	
Flow	1	56.3 ± 11.5	78.7 ± 8.5	97.57
	2	9.8 ± 1.2	34.6 ± 3.8	
	3	1.8 ± 0.1	14.9 ± 2.8	

**Table 7:** Kinetic data for the hydrogenation of aromatic nitro compounds by Cu/CuO/PET TeMs annealed at 140 °C for 5 h [32].

Loaded Catalyst	Investigated Nitro Compound	k, min^−1^	E_a_, kJ mol^−1^	D, %
Cu/CuO/PET TeMs (5 h, 140 °C)	*p*-NP	0.29	39.9	98.1
	*p*-NA	0.26	13.4	90.6
	*p*-NBA	0.30	52.3	83.9

**Table 8:** Composition of iridium plating solutions for electroless deposition on polycarbonate membranes [139].

Stabilizer	Solution 1 (Citrate Stabilized)	Solution 2 (EDA Stabilized)
IrCl_3_·xH_2_O	5 mM	3 mM
EDA	-	18 mM
Na_3_C_6_H_5_O_7_·2H_2_O	20 mM	-
HCl	15 mM	-
NaBH_4_	50 mM (added slowly)	60 mM

**Table 9:** Summary of studies on the decomposition of nitro compounds using composite track-etched membranes (CTeMs).

Type of CTeM	Membrane	Utilized Nitro Compound	Rate Constant, k	Decomposition Degree, D, %	Ea, kJ/mol	Cycles	Refs.
Ag/PC	PC TeM (30 µm, 1 × 10^8^ cm^−2^)	*p*-NP	0.053 s^−1^	-	-	-	[116]
Au/PET	PET TeM (12 µm, 1 × 10^9^ cm^2^)	*p*-NP	0.074 ± 0.02 min^−1^	98.1	-	5	[155]
Ag/PET	PET TeM (12 µm, 1 × 10^9^ cm^−2^)	*p*-NP	0.085 min^−1^	89.8	51.19	3	[29]
Pd/PC	PC TeM (30 µm, 1 × 10^8^ cm^−2^)	*p*-NP	0.06 s^−1^	-	-	-	[156]
Au/PEI-g-DOPA/PET	PEI-*g*-PET TeM (1.5 × 10^8^ cm^−2^)	*p*-NP	0.00409 s^−1^	99% at 40 L m ^−2^ h^−1^ permeation rate	-	11	[30]
Au/PET-Ox	PET TeM (12 µm, 4 × 10^7^ cm^−2^)	*p*-NP	0.0466 ± 0.0031 min^−1^	-	-	-	[117]
Cu/PET	PET TeM (12 µm, 4 × 10^7^ cm^−2^)	*p*-NP	56.3 ± 11.5 × 10^−2^, min^−1^ (flow mode) 5.1 ± 0.4 × 10^−2^, min^−1^ (static mode)	78.7 ± 8.5 (for flow mode) 98.4 ± 4.1 (for static mode)	28.32 (static mode) 97.57 (flow mode)	3	[157]
Cu/CuO/PET	PET TeM (12 µm, 4 × 10^7^ cm^−2^)	*p*-NP	0.29 min^−1^ (reduction of *p*-NP)	98.1 (reduction of *p*-NP)	39.9 (reduction of *p*-NP)	5	[32]
		*p*-NA	0.26 min^−1^ (reduction of *p*-NA)	90.6 (reduction of *p*-NA)	13.4 (reduction of *p*-NA)		
		*p*-NBA	0.30 min^−1^ (reduction of *p*-NBA)	83.9 (reduction of *p*-NBA)	52.3 (reduction of *p*-NBA)		
Ir/PC	PC TeM (25 µm, 1.5 × 10^8^ cm^−2^)	*p*-NP	at 25 °C 0.033 min^−1^ at 35 °C 0.043 min^−1^ at 60 °C 0.082 min^−1^	80	21.3	8	[139]
Ni@Au/PET	PET TeM (12 µm, 4 × 10^7^ cm^−2^)	*p*-NP	0.0017 s^−1^	80	-	5	[103]

**Table 10:** Kinetic data founded through the decomposition of H_2_O_2_ [29].

Cycles	Apparent Constant Rate [k × 10^−3^ min^−1^] H_2_O_2_ Decomp.	Conversion of Initial Reagent H_2_O_2_ Decomp.
1st	16.5	73.53%
2nd	7.0	54.7%
3rd	-	-

**Table 11:** Kinetic data for different Ag/PET/CTeMs for the decomposition of H_2_O_2_ [182].

Deposition Time, min	1 × 10^9^ Ion/cm^2^			4 × 10^7^ Ion/cm^2^		
	t, min	V (O_2_), mL	k, min^−1^	t, min	V (O_2_), mL	k, min^−1^
30	215 ± 27.8	60 ± 1.3	8.7 ± 0.95	185 ± 15	58.6 ± 1.3	12.3 ± 1.49
60	165 ± 17.3	57.3 ± 4.1	15.2 ± 3.4	180 ± 8.7	59 ± 0.9	13.0 ± 3.0
180	193 ± 40.1	60.3 ± 2.3	11.1 ± 1.85	175 ± 8.7	59.53 ± 0.4	12.4 ± 0.38
300	220 ± 20.0	61.7 ± 0.4	9.33 ± 0.83	155 ± 25.0	60.1 ± 0.8	14.9 ± 2.15

**Table 12:** Summary of studies on the decomposition of inorganic compounds using composite track-etched membranes (CTeMs).

Type of CTeM	Polymer Template	Utilized Compound	k, min^−1^	E_a_, kJ/mol	D, %	References
Ag/PET	PET (12 µm, 4 × 10^7^ cm^−2^)	H_2_O_2_	12.3 ± 1.49 (for 30 min Ag deposition time)	34.35	-	[182]
			14.9 ± 2.15 (for 300 min Ag deposition time)	39.25	-	
Cu_Asc/PET	PET (12 µm, 4 × 10^7^ cm^−2^)	K_3_[Fe(CN)_6_]	0.4	7.1	94.4	[121]
Cu_DMAB/PET	PET (12 µm, 4 × 10^7^ cm^−2^)	Cr (IV)	0.017	10.8	99.88	[140]
Cu_Gly/PET			0.156	35.96	99.56	
Cu_CHOH/PET			0.249	37.00	41.04	

## Data Availability

The data presented in this study are available on request from the corresponding authors.
